# Reusing water, reshaping nitrogen: Unravelling nitrogen dynamics in soils receiving treated wastewater

**DOI:** 10.1002/jeq2.70301

**Published:** 2026-11-05

**Authors:** Vasileios A. Tzanakakis, Georgios Giannopoulos, A. G. Capodaglio, A. N. Angelakis

**Affiliations:** ^1^ Department of Agriculture, School of Agricultural Science Hellenic Mediterranean University Iraklion Greece; ^2^ School of Agriculture Aristotle University of Thessaloniki Thessaloniki Greece; ^3^ Department of Civil Engineering & Architecture University of Pavia Pavia Italy; ^4^ Hellenic Agricultural Organization (ELGO DIMITRA), National Foundation for Agricultural Research, Institute of Iraklion Iraklion Greece; ^5^ Hellenic Union of Municipal Enterprises for Water Supply and Sewerage Larisa Greece

## Abstract

Soils are increasingly receiving treated wastewater (TWW) to support crop production and protect natural resources. However, besides water, various inputs, such as soil nutrients (salts), organic carbon, and various contaminants of emerging concern, including antibiotics, antibiotic resistance genes, pharmaceutical compounds, heavy metals, and microplastics, can be delivered. This review aims at (a) an integrated and mechanistic synthesis of current knowledge on potential impacts of TWW irrigation on key soil nitrogen turnover processes, such as nitrogen mineralization, nitrification, denitrification, and ammonia volatilization, and their drivers, and (b) identification of key knowledge and conceptual uncertainties to support future studies. Across the synthesis, no single, uniform TWW effect emerges. In fact, outcomes are consistently conditional on several co‐factors, such as treatment level, irrigation duration, soil type, and plant species, with the latter two sometimes exerting effects exceeding those of water quality itself. TWW alters soil conditions through substrate availability, redox dynamics, soil acidity and salinity, and contaminant exposure, but the mechanistic evidence linking specific drivers to specific nitrogen‐process outcomes is itself uneven. Identified knowledge and methodological gaps suggest long‐term field studies across TWW regimes, soil types, and climates; designs capable of disentangling co‐varying drivers at field scale; and laboratory studies addressing contaminant‐specific impacts, microbial functional persistence, and potential decoupling between nitrogen and carbon dynamics, an effect already evident, though unexplored as a general phenomenon, within the reviewed literature. Overall, this review offers an integrated, system‐level framework to support the evidence‐based management of environmentally and climate‐vulnerable soils receiving reclaimed or even conventional irrigation water.

## Plain Language Summary

More farms are using treated wastewater to irrigate crops, saving freshwater, and reusing a resource that would otherwise go to waste. But this water carries more than just water and nutrients, and it also brings salts, organic matter, and modern pollutants like antibiotic residues, resistant bacteria, heavy metals, and microplastics. This review summarizes what is known about how such irrigation affects soil nitrogen cycling, the processes that make nitrogen available to plants or release it into air and groundwater, and identifies what remains poorly understood. The main finding is that there is no single, simple effect. Outcomes depend heavily on treatment level, irrigation duration, soil type, and crop, sometimes more than on water quality itself. Some of these connections are well understood; many are not. We outline priority research needs, including better‐designed field studies and closer attention to how soil nitrogen and carbon cycling may shift independently of each other.

**Abbreviations**
AOA: ammonia‐oxidizing archaea
AOB: ammonia‐oxidizing bacteria
ARGs: antibiotic resistance genes
BOD_5_
: 5‐day biochemical oxygen demand
CECs: contaminants of emerging concern
COD: chemical oxygen demand
Comammox: complete ammonia oxidation
DOC: dissolved organic carbon
EC: electrical conductivity
EDCs: endocrine‐disrupting chemicals
EU: European Union
GHG(s): greenhouse gas(es)
MAOM: mineral‐associated organic matter
NOB: nitrite‐oxidizing bacteria
OM: organic matter
PFAS: polyfluoroalkyl substances
POM: particulate organic matter
PW: primary‐treated wastewater
RW: reclaimed water
SAT: soil aquifer treatment
SOC: soil organic carbon
SOM: soil organic matter
TKN: total Kjeldahl nitrogen
TN: total nitrogen
TWW: treated wastewater
UF: ultrafiltration
UV: ultraviolet
WFPS: water‐filled pore space
WTP (‐A, ‐B, ‐C): wastewater treatment plant (plant codes as in the original study)
WWTP(s): wastewater treatment plant(s)

## 1 INTRODUCTION

Treated wastewater (TWW) from wastewater treatment plants (WWTPs), supported by advances in treatment technology, is increasingly applied in growing populations and/or water‐scarce areas, like the Mediterranean region, to support water supply, agricultural production, and protect natural resources (Mainardis et al., 2022; Tzanakakis et al., 2023). This practice is compatible with the global transition to the circular economy (United Nations‐Water [UN‐WATER], 2017) and the achievement of sustainable development goals (e.g., Target 6.3, Clean Water and Sanitation, and others) (Tzanakakis et al., 2023). In the European Union (EU), increasing wastewater reuse has become a priority to alleviate water scarcity across Member States, consistent with the current recast of the Urban Wastewater Treatment Directive (EU, 1991), the Water Framework Directive (European Parliament and Council, 2000), and other policies and strategies dealing with the circular economy and adaptation to climate change (European Commission [EC‐COM], 2019, 2020); across the EU members, irrigation uses represent 52% of the total tertiary treated water, while industry and non‐potable urban applications represent 19.3% and 8.3%, respectively (Lautze et al., 2014). Israel and Singapore have achieved water reuse percentages up to 24% and 30% of their total water supply, respectively (X. C. Wang et al., 2015), whereas in China, reused TWW reaches 3.76 billion m^3^/year (Y. Sun et al., 2016).

At the global scale, 13.4% of the energy demand for highly energy‐demanding synthetic fertilizers could potentially be offset by recovered nutrients from TWW, while reducing WWTPs’ energy requirements for their removal (Qadir et al., 2020). At the EU level, TWW‐recovered nitrogen could meet up to 14% of fertilization requirements (Kehrein et al., 2020). In China, estimates for nitrogen loading in reused effluent are set at 2.05 × 10^5^ and reused organics (as chemical oxygen demand [COD]) at 2.12 × 10^6^ Mg/year (Y. Sun et al., 2016). Despite the nitrogen value to TWW‐irrigated agroecosystems, potential nitrogen loss may contribute to the degradation of subsurface/surface water resources and to undesired nitrous oxide (N_2_O) emissions (Lal et al., 2015; S. Lu et al., 2016; Tzanakakis et al., 2023), a powerful greenhouse gas (GHG); nitrogen loss is a central challenge for agricultural soils in general (Grzebisz & Niewiadomska, 2024; L. Liu et al., 2020) related to diverse nitrogen sources (Brzezinska et al., 2006; Chauhan et al., 2025; Speir, 2002) and the complexity of nitrogen processes occurring within ecosystems and across the soil–water–plant–atmosphere continuum (Iqbal et al., 2025; Robertson & Groffman, 2024); the overall anthropogenic N_2_O emissions (approximately 50% of the total) are estimated at the global scale to total 9.7 million metric tons/year globally (L. Liu et al., 2020). Such losses are estimated to amount to around $200 billion worth of nitrogen resources wasted annually. Given that nitrogen in the natural cycle relies on a plethora of biochemical and physical processes in the soil–water–plant–atmosphere system, efficient nitrogen management to support crop production and minimize the adverse impacts on the environment in TWW‐ or freshwater‐irrigated lands remains a central challenge.

Despite advances in wastewater treatment, TWW still may carry unwanted pollutants/contaminants of emerging concern (CECs), such as pathogens, heavy metals, antibiotics and antibiotic resistance genes (ARGs), polyfluoroalkyl substances (PFAS), endocrine‐disrupting chemicals (EDCs) (e.g., triclosan and bisphenol), and micro‐ and nanoplastics (Al Salah et al., 2019; Arditsoglou & Voutsa, 2010; Giannopoulos et al., 2026; Karkman et al., 2018; Ofiera et al., 2025; Pazda et al., 2019; Sabri et al., 2018; Voutsa et al., 2006; Zerva et al., 2018) originating from inadequately processed urban discharges (Tan & Shuai, 2015; Zerva et al., 2018). These contaminants can interfere with the soil matrix and crops, risking human health, biodiversity, and ecosystem quality and functioning (Adegoke et al., 2018; Roose‐Amsaleg & Laverman, 2016; Xie et al., 2019). Antibiotic resistance has been recognized by the WHO (World Health Organization) as one of the most critical public health threats of the 21st century (Pazda et al., 2019), and TWW irrigation is a documented transmission pathway, with ARGs and resistant bacteria detected in water designated for agricultural reuse (Cadena‐Aponte et al., 2025; Macrì et al., 2024). As the reuse of TWW is increasing, these issues, along with the potential impact on critical nitrogen cycling processes, should be efficiently addressed through evidence‐based characterization of TWW quality by treatment level, identification of the soil and microbial drivers most reliably linked to nitrogen‐cycle outcomes, and targeted research addressing the specific knowledge gaps this constitutes; these are the aims pursued in the present review.

The objectives of this review are to (a) provide an integrated and mechanistic synthesis of current knowledge on potential impacts of TWW irrigation on critical biochemical nitrogen processes within the soil–water–plant–atmosphere system, and (b) identify and highlight the key knowledge, methodological, and conceptual gaps to address in future studies. For the review, a thorough literature review was undertaken in mainstream databases (e.g., Scopus and Web of Science) based on selected keywords combinations, such as: treated wastewater; treated wastewater reuse; treated wastewater irrigation; reuse of reclaimed water; soil microorganisms; nitrogen mineralization; enzymes; nitrification; ammonia oxidation; AOA; AOB; NOB; comammox; nitrifier denitrification; heterotrophic denitrification; CECs; ARGs; EDCs; antibiotics; heavy metals; microplastics; soil salinity; plant species; nitrogen plant uptake; nutrient use efficiency (NUE); water use efficiency (WUE); carbon cycling; coupling of carbon and nitrogen cycling; carbon sequestration.

## 2 CURRENT TRENDS AND CHALLENGES IN TWW QUALITY AND AGRICULTURAL REUSE

### 2.1 TWW quality and regulatory frameworks

#### 2.1.1 Nitrogen

Nitrogen is originally present in wastewater in the form of organic nitrogen and ammonia/ammonium, resulting from the initial hydrolysis of urea and human/animal excreted proteins. There is high variability regarding the TWW nitrogen content, differing substantially with source and treatment level. Τhere is high variability in total nitrogen (TN) content across the examples of TWW sources reviewed in this review study (Table 1). For example, aw or under‐TWW compiled historical sources report TN in the range of 29.3–53 mg L^−^^1^ (Aguilar‐Rangel et al., 2024) (compiled literature range), whereas secondary‐treated effluents also show comparably a wide range of variation, from 45–53 mg L^−^^1^ TN (W. Guo et al., 2018; Werheni Ammeri et al., 2023) and 60–73 mg L^−^^1^ TKN (total Kjeldahl nitrogen) (Feki et al., 2025) to 19.7 mg L^−^^1^ TKN (J. F. Wang et al., 2007) and 23 mg L^−^^1^ NH_4_^+^‐N alone at a stabilization‐pond site (de Paula et al., 2009); notably, advanced treatment does not guarantee the lowest TN (Mola et al., 2024). This underscores that treatment level alone may not be a reliable predictor of TN load without knowing the specific treatment processes and influent‐specific characteristics.

**TABLE 1:** Treated wastewater (TWW) physicochemical characteristics as affected by treatment level.

Treatment level	Organic C (BOD/COD)	Nitrogen (TN/NH_4_^+^/NO_3_^−^)	Ph	EC	Reference
Raw/under‐treated	Not reported	Not reported	6.92–7.70	0.30–2.32	Prasad et al., 2026
Not stated (STP effluent)	Not reported (P: 0.56–5.91; K: 9.33–23.1; S: 7.71–22.9 mg/L)	Not reported	5.8–6.5	1.36–2.88	Rattan et al., 2005
Primary/basic (settling/oxidation ponds)	Not reported	NO_3_^−^: 15.2–23.5 (mean 18.1)	7.00–7.57	1.32–1.60	Rezapour et al., 2019
Secondary (Lysimeter site, baseline TWW)	BOD 16; COD 55	Not reported	8.7	2.1	Heinze et al., 2014
Tertiary (further on‐site processing)	BOD 18; COD 63	Not reported	8.2	1.9	Heinze et al., 2014
Advanced/UF (ultra‐filtration)	BOD 8.1; COD 55	Not reported	7.8	2.0	Heinze et al., 2014
Secondary (Batsra site, same paper)	BOD 10; COD 25	Not reported	7.7	1.3	Heinze et al., 2014
Secondary (Gaza site, same paper)	BOD 96; COD 242	Not reported	8.4	2.2	Heinze et al., 2014
Secondary	BOD: 123–700 (1984–2009)/180 (2016–2018); COD_5_: 37–220 (1984–2009)/610 (2016–2018)	NTK 60–73; NH_4_^+^‐N 48 (2016–2018); NO_3_^−^ 0.35–50 (1984–2009)/ND (2016–2018)	7.1–8.7/7.31–7.46	4–7.7/5.40–5.72	Feki et al., 2025
Secondary (Sfax WWTP, qualitative only)	“Always exceeds” NT106.03 (no numeric value given)	Not reported	“always alkaline” (no range given)	Salinity 3.5–5 g/L (not dS/m)	Belaid et al., 2019
Secondary	BOD_5_ 83.4; COD 212.6	Kjeldahl‐N (TKN) 19.7	7.06	Salinity 782 mg/L (not dS/m)	Wang et al., 2007
Secondary	COD_m__n_ 13.37 (vs. 7.86 clean‐water control)	TN 45.14; NH_4_^+^‐N 11.11; NO_3_^−^‐N 20.62	7.4	1.7	W. Guo et al., 2018
Raw/untreated (compiled literature range, 1985–2018, not primary data of the citing paper)	TOC 35–188 (1997–98); DOC 15–80 (2011); DOC 180 (2017–18, diff. source)	TN 29.3–53; NH_4_^+^‐N 17.5–33.5; NO_3_^−^‐N 0–3.3	6.7–8.9	930–2,264 µS/cm (≈0.93–2.26 dS/m)	Aguilar‐Rangel et al., 2024 (compiled)
Secondary	BOD_5_ 126.0; COD 575.65	N (Kjeldahl) 53.15; NH_4_^+^ 13.43; NO_3_^−^ 2.06; Phosphates 4.62	7.72	5.31	Werheni Ammeri et al., 2023
Primary (septic tank)	BOD 348	TKN 105; NH_4_^+^‐N 89; NO_3_^−^‐N 1.1	7.34	2.07	Tzanakakis et al., 2011
Secondary (stabilization ponds − 3 anaerobic + 3 facultative)	ST (total solids) 127.8 (not BOD/COD)	NH_4_^+^‐N 23; NO_3_^−^‐N 0.30	7.5	0.85	de Paula et al., 2009
Secondary (activated sludge)	Not reported (SS 17.0 mg/L)	NH_4_^+^‐N 8.67; NO_3_^−^‐N 5.42	7.8	2.32	Adrover et al., 2012
Advanced/Class A (UASB + constructed wetland + UV)	BOD_5_ 7.5; tCOD 35.2	TN 66.1; NH_4_^+^‐N 5.7; NO_3_^−^‐N 47.7 (>FAO limit of <30)	6.8	1.19 (>FAO limit of <0.7)	Mola et al., 2024
Not stated	RW: BOD 40.32 ± 8.79; COD 79.63 ± 26.87 (vs. GW: BOD 11.60; COD 15.10)^a^	RW: TN 28.10 ± 19.85 (vs. GW: 1.36)	not reported	not reported (Total salt 720.20 ± 136.48 mg/L vs. GW 334.00)	S. Lu et al., 2016
Tertiary (original, pre‐adjustment TWW)	DOC 13 (no BOD/COD given)	NH_4_^+^ 1.61 mmolc/L (not mg/L, see note)	7.2	1.74	Ibekwe et al., 2018
Secondary (pre‐2007 upgrade)	BOD 25–80; COD 173–300	TN 30–48; NH_4_^+^‐N 25.0–40.0; NO_3_^−^‐N 0.1–1.7	7.1–7.5	1.8–2.2	Elifantz et al., 2011
Secondary‐upgraded (post‐2007)	BOD 5–10; COD 52	TN 7.0–10.0; NH_4_^+^‐N 5.0–7.5; NO_3_^−^‐N 0.1–1.0	7.4	1.5	Elifantz et al., 2011
Secondary (El Bobar UWWTP effluent, pre‐tertiary)	not reported	NH_4_^+^‐N 69.2 ± 16.4	not reported	not reported (turbidity 7.4 ± 1.8 NTU)	Cadena‐Aponte et al., 2025
Tertiary (sand filtration + chlorination, final RW for irrigation)	DOC 19.2 ± 4.4 (no BOD/COD given)	NH_4_^+^‐N 74.9 ± 16.4	7.6 ± 0.2	3.0 ± 0.3 (upper limit of recommendation for the irrigated crop)	Cadena‐Aponte et al., 2025

Abbreviations: BOD_5_, 5‐day biochemical oxygen demand; BOD, biochemical oxygen demand; COD, chemical oxygen demand; EC, electrical conductivity; DOC, dissolved organic carbon; FAO, Food and Agriculture Organization of the United Nations; GW, groundwater; ND, not detected; RW, reclaimed water; SS, suspended solids; STP, sewage treatment plant; tCOD, total chemical oxygen demand; TKN/NTK, total Kjeldahl nitrogen (French: azote total Kjeldahl; = TKN); TN, total nitrogen; TOC, total organic carbon; UASB, upflow anaerobic sludge blanket; UV, Ultraviolet; UWWTP, urban wastewater treatment plant.

^a^ Compared with groundwater rather than with another treatment level, unlike other rows in this table.

Nitrate pollution is widespread around the globe (Abascal et al., 2022); in the EU, the Nitrates Directive (91/676/EEC [European Economic Community]) has been effective since 1991 to protect water quality from pollution by agricultural sources, promoting good farming practices, and setting nitrates limitation to 50 mg/L: although harmless per se, nitrates are, in particular conditions, reduced to nitrite in the human body, possibly inducing methemoglobinemia (also known as “blue baby syndrome”), a condition in which oxidation of hemoglobin to methemoglobin impairs blood oxygen transport capacity once methemoglobin levels exceed 5%–10% of total hemoglobin. Notably, however, Regulation (EU) 2020/741 defines its four reclaimed‐water quality classes (A–D) around pathogen indicators, 5‐day biochemical oxygen demand (BOD5), total suspended solids, and turbidity, not nitrogen species—so two waters in the same regulatory quality class for irrigation can differ several‐fold in N loading depending on whether upstream nitrogen removal was independently required or installed. For the purpose of the review study, the physicochemical characteristics of TWW, including organic carbon, nitrogen, pH, and electrical conductivity (EC), under different treatment levels are shown in Table 1.

#### 2.1.2 Contaminants of emerging concern

Throughout this review, “CECs” refers exclusively to contaminants of emerging concern; cation exchange capacity, an unrelated soil property discussed elsewhere in this review, is always spelled out in full to avoid ambiguity between the two. Despite advances in wastewater treatment, CECs, including pathogens, antibiotics and ARGs, EDCs, PFAS, heavy metals, and micro(nano) plastics, cannot be eliminated and may undergo further transformation into metabolic byproducts (Table 2); TWW may carry CECs at the ng/L level, challenging their detection (Capodaglio, 2017; Giannopoulos et al., 2026). At these levels, no acute risk is present, but accumulation in soil and crops may be hazardous in the long term (Denora et al., 2023). Removal of CECs is compound‐specific and does not scale simply with treatment level (Koumaki et al., 2025). This compound‐class‐dependent pattern is consistent with findings from a 445‐field survey in Israel (Ben Mordechay et al., 2021). Under controlled dosing conditions simulating tertiary treatment, compound‐specific soil retention has also been demonstrated (Denora et al., 2023). ARGs in the irrigation water itself, upstream of soil deposition, persist despite treatment, correlating with Cu, Ni, and Se concentrations (Macrì et al., 2024), while tertiary treatment (sand filtration + chlorination) at a Spanish facility reduced six monitored ARGs marginally, indicating the chlorination's inefficacy against ARGs specifically (Cadena‐Aponte et al., 2025). Notably, ARG concentrations can increase further downstream of the treatment plant itself, within on‐farm storage reservoirs and drip‐irrigation tubing (Cadena‐Aponte et al., 2025), indicating that treatment level alone does not determine final ARG exposure. Examples of certain CECs’ content across different studies and treatment scales are shown in Table 2.

**TABLE 2:** Contaminants of emerging concern (CECs) in effluents.

Contaminant category	Representative compounds	Treatment level	Concentration range	Key observations	Reference
Mixed CECs (antibiotics, antifungals, anti‐inflammatories, beta‐blockers, antidepressants)	Clarithromycin, trimethoprim, sulfamethoxazole, fluconazole, clotrimazole, diclofenac, ketoprofen, naproxen, metoprolol, carbamazepine	Tertiary (rapid sand filtration + UV)	Experimentally spiked: 200 µg/L (TWWx1) and 600 µg/L (TWWx3) per compound—not ambient concentrations*	Ferrandina, Italy, lysimetric plots; compound‐specific soil retention (up to 100% for climbazole; naproxen/diclofenac fully degraded)	Denora et al., 2023
Corrosion inhibitor/anticonvulsants/antiparasitics/antidepressants/antimicrobials	Benzotriazole, carbamazepine, lamotrigine, thiabendazole, venlafaxine, sulfamethoxazole, trimethoprim	Mixed (activated sludge/+sand filtration/+reservoir residence/+SAT, pooled)	Ambient field concentrations, for example, benzotriazole median 539.7 ng/L (max 26,238); carbamazepine median 270.1 ng/L (max 9354); thiabendazole median 26.4 ng/L (max 10,992)	445 fields, Israel, 2017–2019, 318 water samples; SAT treatment showed the lowest cumulative CEC load in a separate cluster analysis (companion paper)	Ben Mordechay et al., 2021, ^a^
Antibiotics (mixed classes)	Tetracycline, sulfamethoxazole, ofloxacin, roxithromycin	Secondary (synthetic water simulating secondary MWTP effluent)	Experimentally spiked: 0, 10, 30, 100, 500 µg/L per compound—not ambient*	4‐year rice‐paddy microcosms; soil concentration proportional to water concentration; paired ARG/heavy‐metal‐resistance‐gene metagenomics	Zheng et al., 2023
Mixed CECs (antihypertensives, antihistamines, antibiotics, psychoactive drugs, benzotriazoles, UV filter)	Telmisartan, irbesartan, metoprolol (antihypertensives); fexofenadine, cetirizine (antihistamines); clarithromycin, trimethoprim (antibiotics); gabapentin, carbamazepine (psychoactive); benzotriazoles; PBSA	Not stated (standard municipal WWTP)	Ambient: listed classes > 100 ng/L; PBSA specifically 240–2100 ng/L (seasonal)	Czech Republic; 59/75 screened compounds detected in effluent; seasonal fluctuation tied to consumption patterns; Arenosol showed higher leaching than Cambisol	Kodešová et al., 2024, ^b^
Mixed CECs—anticonvulsants, antihypertensives, sweeteners, stimulants	Gabapentin, valsartan, carbamazepine TPs, acesulfame, caffeine	Advanced/Class A (UASB + 2‐stage constructed wetland + UV, meets EU 2020/741 Class A)	Gabapentin: mean 5062, max 6978 ng/L; Valsartan: mean 4824, max 10,161 ng/L; Acesulfame: mean 2907, max 4677 ng/L; Caffeine: mean 339, max 1151 ng/L	Lesvos, Greece; several compounds (gabapentin, carbamazepine TPs) showed negative removal—concentration increased after treatment (TP reversion/deconjugation)	Koumaki et al., 2025
Antimicrobial agents	Sulfamethoxazole, trimethoprim	Advanced/Class A (as above)	Sulfamethoxazole: mean 68.2, max 273 ng/L (94% removal); Trimethoprim: below LOD in reclaimed water (100% removal)	Same site as above; antibiotics show high removal, contrasting with the anticonvulsant/sweetener group—illustrates compound‐class‐dependent treatment efficacy.	Koumaki et al., 2025
Mixed pharmaceuticals (analgesics, antibiotics, antidiabetics, lipid regulators)	Naproxen, sulfamethoxazole, metformin, carbamazepine, ciprofloxacin, gemfibrozil	Raw/untreated (compiled literature range, 1985–2018, not primary data of the citing paper)	∼0.02–89.7 µg/L across compounds (e.g., naproxen 4.8–18.6; sulfamethoxazole 4.5–6.6; metformin 39–89.7)	Compiled historical literature at the same untreated‐wastewater site (Mezquital Valley, Mexico), not primary data of the citing paper	Aguilar‐Rangel et al., 2024
ARGs/ARB (antibiotic resistance)	*sulII* gene (sulfonamide resistance); sulfamethoxazole‐resistant bacteria (SRB); ampicillin‐resistant bacteria (AmRB); tetracycline‐resistant bacteria (TRB)	Secondary + sand filtration/H_2_O_2_/UV (step F = WWTP effluent for agricultural reuse)	*sulII*: 2.57–4.34 log gene copies/mL (steps D/E/F); SRB: 1.70–4.77 log CFU/mL (D/E/F); ampicillin (antibiotic itself, not ARB) mean 0.056 ng/L at step F	Italy, 280,000 p.e., 5.5 million m^3^/year reused for pasture/alfalfa/corn/tomato/vineyard irrigation; significant reduction influent→effluent (*p* < 0.05) but still detectable in reuse‐designated water; TRB/AmRB significantly correlated with Cu, and TRB/total ARB with Se (*r* > 0.6, *p* < 0.001); *sulII* also positively correlated with Ni (r/p not specified in text); no correlation found with antibiotic concentration itself.	Macrì et al., 2024
Mixed antibiotics (fluoroquinolones, macrolides, sulfonamides)	Ciprofloxacin, levofloxacin/ofloxacin, azithromycin, sulfamethoxazole, trimethoprim	Tertiary (sand filtration + chlorination, real‐scale, Almería, Spain)	Total load: 4847 ± 1413 ng/L (secondary effluent, UWW) → 2050 ± 813 ng/L (post‐chlorination, 58% removal, *p* = 0.0005) → 1981 ± 1018 ng/L (after storage, no further significant change)	Highly compound‐specific removal: ciprofloxacin 100% removed by chlorination; azithromycin and levofloxacin showed ∼0% removal (unchanged or slightly increased); sulfamethoxazole 56% removed; trimethoprim 53% removed	Cadena‐Aponte et al., 2025
ARGs (antibiotic resistance genes)	16S rRNA, *intI1*, *blaCTX‐M32*, *sul1*, *blaTEM*, *qnrS*	Tertiary (sand filtration + chlorination, real‐scale, Almería, Spain)	Log units/100 mL, secondary effluent (UWW) → final reclaimed water (RW‐R): 16S rRNA 10.46→9.99; *intI1* 9.52→8.80; *blaCTX‐M32* 8.08→7.53; *sul1* 7.91→7.08; *blaTEM* 7.35→6.81; *qnrS* 6.20→5.72	Only modest reduction throughout (0.47–0.83 Log), statistically significant for just 3 of 6 targets (16S rRNA, *blaCTX‐M32*, *sul1*); chlorination confirmed ineffective at removing ARGs. In one of four greenhouse drip‐irrigation systems monitored downstream, *intI1*, *blaTEM* and *qnrS* concentrations increased further (+15.7%, +18.5%, +11.3%, respectively) relative to the storage reservoir, attributed to bacterial regrowth/biofilm in distribution pipwork	Cadena‐Aponte et al., 2025

Abbreviations: ARB, antibiotic‐resistant bacteria; EU, European Union; LOD, limit of detection; MWTP, municipal wastewater treatment plant; PBSA, 2‐phenylbenzimidazole‐5‐sulfonic acid; SAT, soil aquifer treatment; TP, transformation product; UASB, upflow anaerobic sludge blanket; UV, ultraviolet; UWW, urban wastewater.

^a^ Companion publication to Ben Mordechay et al. (2022), also cited in this review, from the same research group and survey.

^b^ Comparison based on soil type (Arenosol vs. Cambisol) rather than on treatment level, unlike other rows in this table.

The potential impacts of CECs on nitrogen cycling processes are still challenging issues in need of elucidation, mainly concerning impacts on soil microorganisms and their activities, crop responses, and soil–crop interactions (Kwak et al., 2017; Yakamercan et al., 2025). Recently, the EU released water reuse guidelines (European Commission [EC‐COM], 2018) in which pharmaceuticals (antibiotics) and antimicrobial resistance are indicated as being of great concern because of their potential introduction into the trophic chain and possible harm to biodiversity and human health (Pazda et al., 2019). However, as of today, the criteria for these substances have not yet been elaborated. The WHO has recognized antibiotic resistance as one of the most critical public health issues for the 21st century (Pazda et al., 2019).

Micro‐ and nanoplastics are another category of emerging pollutants in TWW‐irrigated soils (Aydin et al., 2025). Although conventional treatment in WWTPs can remove substantial quantities of microplastics (up to 95% of those between 100 µm and 5 mm), removal of nanoplastics (between 1 nm and 1 µm) is usually very low and difficult to assess (Capodaglio, 2024). The estimated removal efficiency scales directly with the treatment level: 70% for primary, 85% for secondary, 95% for tertiary, and up to 99% for advanced treatment such as sand filtration or membranes. Microplastic concentrations in the irrigation water itself decrease substantially with treatment, but absolute residual loads vary by up to three orders of magnitude across facilities, even within the same treatment category (Avilés et al., 2025; Çolakoğlu et al., 2026; Flores et al., 2026; Lv et al., 2025), indicating that treatment level alone is a poor predictor of the microplastic loading. Besides TWW irrigation, nanoplastics can be transferred to soil‐water systems via atmospheric wet and dry deposition. Examples of microplastic concentrations in TWW are shown in Table 3.

**TABLE 3:** Microplastics in wastewater.

Treatment level	Particle count (MPs/m^3^, inlet → outlet)	Mass (µg/L, outlet)	Reference
Secondary only (WTP‐C)	Σ31,093 → Σ12,467 (60% removal)	54.1	Flores et al., 2026
Tertiary (sand filtration + ozonation, WTP‐B)	Σ12,433 → Σ112 (99% removal)	0.4	Flores et al., 2026
Advanced/tertiary (ultrafiltration, WTP‐A)	601–2,934 → 89–399 (0.09–0.39 MPs/L outlet; 89% removal)	∼11 → ∼2 (82% removal)	Flores et al., 2026
Advanced (modified Bardenpho‐MBR, Chengdu Cooperative WWTP)	517,000 → ∼27,900^a^ (94.6% removal)	Not reported	Lv et al., 2025
Tertiary (AAO + deep‐bed filter + UV, Unity Town WWTP)	177,000 → ∼15,900^a^ (91% removal)	Not reported	Lv et al., 2025
Tertiary (AAO + activated coke + deep‐bed filter, no UV stated; Airport Phase I WWTP)	332,000 → 28,000 (91.6% removal; HRT 8 h)	Not reported	Lv et al., 2025
Tertiary (AAO + deep‐bed filter + UV, Hedong WWTP, Jianyang)	410,000 → 44,000 (89.3% removal, lowest of four; HRT only 6 h)	Not reported	Lv et al., 2025
Not stated (industrial zone WWTP—textile, plastic recycling, furniture sectors)	∼433,000^a^ → 52,000 (88% removal)	Not reported	Çolakoğlu et al., 2026
Full train (pretreatment → secondary → tertiary ring filtration + UV; total water reuse + agricultural sludge application, Murcia, Spain)	not reported (this source measured mass, not particle count)	26,580 (pretreatment) → 1080 (secondary) → 610 (tertiary, final reuse water)	Lozano Avilés et al., 2025

Abbreviations: AAO, anaerobic–anoxic–oxic; HRT, hydraulic retention time; MBR, membrane bioreactor; MPs, microplastics; UV, ultraviolet.

^a^ Value not reported explicitly in the cited source; calculated by the authors from the other reported concentration and the reported removal percentage.

Heavy metal concentrations in TWW irrigation water are consistently low relative to FAO (Food and Agriculture Organization of the United Nations)/WHO guideline values (Ayers & Westcot, 1994) across the treatment levels reviewed in this study (Table 4), from raw/under‐treated sources (Aguilar‐Rangel et al., 2024; Prasad et al., 2026) through primary (Rezapour et al., 2019) to secondary and advanced treatment (Belaid et al., 2019; de Paula et al., 2009; Feki et al., 2025; W. Guo et al., 2018; Macrì et al., 2024; Mola et al., 2024), with values for Cu, Ni, Cd, Pb, Zn, Fe, and Mn typically an order of magnitude or more below their respective limits regardless of treatment level. These guideline values were independently corroborated across five geographically distinct studies (de Paula et al., 2009; W. Guo et al., 2018; Rattan et al., 2005; Rezapour et al., 2019; J. F. Wang et al., 2007), reinforcing their reliability as a reference standard.

**TABLE 4:** Heavy metals in wastewater.

Parameter	Raw/primary (mg/L)	Secondary/treated (mg/L)	Int. irrigation standard (FAO/WHO)	References
Cr	0.013–0.037 (Prasad et al., 2026, raw/under‐treated); 0.0005–0.112 (Aguilar‐Rangel et al., 2024, raw/untreated)	0.007–1.455 (Feki et al., 2025); 0.05–1.1 (Belaid et al., 2019); 0.02 (Wang et al., 2007); 0.02008 (W. Guo et al., 2018); ND (de Paula et al., 2009); 0.000394 (Macrì et al., 2024, effluent for reuse)	0.10	Aguilar‐Rangel et al., 2024; Belaid et al., 2019; Feki et al., 2025; Prasad et al., 2026; Wang et al., 2007; de Paula et al., 2009; W. Guo et al., 2018, 2010; Macrì et al., 2024
Ni	0.020–0.084 (Prasad et al., 2026); 0.04–0.14 (Rezapour et al., 2019, primary); 0.086–0.95 (Aguilar‐Rangel et al., 2024, raw/untreated)	0.02–0.13 (Feki et al., 2025); 0.039–0.067 (Rattan et al., 2005); 0.018–0.13 (Belaid et al., 2019); ND (de Paula et al., 2009); 0.03 (Mola et al., 2024, advanced); 0.00488 (Macrì et al., 2024, effluent for reuse)	0.20	Aguilar‐Rangel et al., 2024; Belaid et al., 2019; de Paula et al., 2009; Feki et al., 2025; Prasad et al., 2026; Rattan et al., 2005; Rezapour et al., 2019, 2010; Macrì et al., 2024; Mola et al., 2024
Cd	0.002–0.051 (Prasad et al., 2026); 0.11–0.19 (Rezapour et al., 2019, primary); 0.005–0.05 (Aguilar‐Rangel et al., 2024, raw/untreated)	ND–0.07 (Feki et al., 2025); 0.0012–0.0024 (Rattan et al., 2005); 0.00333 (W. Guo et al., 2018); ND (de Paula et al., 2009); <0.01 (Mola et al., 2024, advanced); <0.000018 (Macrì et al., 2024, effluent for reuse, below LOQ)	0.01	Aguilar‐Rangel et al., 2024; de Paula et al., 2009; Feki et al., 2025; W. Guo et al., 2018; Prasad et al., 2026; Rattan et al., 2005; Rezapour et al., 2019, 2010; Macrì et al., 2024; Mola et al., 2024
Pb	0.006–0.049 (Prasad et al., 2026); 0.47–0.96 (Rezapour et al., 2019, primary); 0.021–0.30 (Aguilar‐Rangel et al., 2024, raw/untreated)	0.001–0.37 (Feki et al., 2025); 0.022–0.067 (Rattan et al., 2005); not detected (Wang et al., 2007); <0.05 (Mola et al., 2024, advanced); 0.000208 (Macrì et al., 2024, effluent for reuse)	5.0	Aguilar‐Rangel et al., 2024; Feki et al., 2025; Prasad et al., 2026; Rattan et al., 2005; Rezapour et al., 2019; Wang et al., 2007; Macrì et al., 2024; Mola et al., 2024
Cu	0.52–0.73 (Rezapour et al., 2019, primary); 0.012–0.15 (Aguilar‐Rangel et al., 2024, raw/untreated)	<0.01–0.123 (Feki et al., 2025); 0.009–0.116 (Rattan et al., 2005); 0.06–0.16 (Belaid et al., 2019); 0.7 (Wang et al., 2007); 0.02 (W. Guo et al., 2018); 0.002 (de Paula et al., 2009); 0.000502 (Macrì et al., 2024, effluent for reuse)	0.20	Aguilar‐Rangel et al., 2024; Belaid et al., 2019; Feki et al., 2025; Rattan et al., 2005; Rezapour et al., 2019; Wang et al., 2007; de Paula et al., 2009; W. Guo et al., 2018, 2010; Macrì et al., 2024
Zn	0.77–0.96 (Rezapour et al., 2019, primary); 0.018–0.46 (Aguilar‐Rangel et al., 2024, raw/untreated)	0.01–0.484 (Feki et al., 2025); 0.006–0.151 (Rattan et al., 2005); 0.1–0.27 (Belaid et al., 2019); 1.3 (Wang et al., 2007); 0.02 (de Paula et al., 2009); 0.13 (Mola et al., 2024, advanced); 0.0154 (Macrì et al., 2024, effluent for reuse)	2.0	Aguilar‐Rangel et al., 2024; Belaid et al., 2019; Feki et al., 2025; Rattan et al., 2005; Rezapour et al., 2019; Wang et al., 2007; de Paula et al., 2009; Macrì et al., 2024; Mola et al., 2024
As	0.005–0.76 (Aguilar‐Rangel et al., 2024, raw/untreated)	0.000293 (Macrì et al., 2024, effluent for reuse)	0.10	Aguilar‐Rangel et al., 2024; Macrì et al., 2024
Fe	not reported	<0.013–1.69 (Feki et al., 2025); 0.639–3.793 (Rattan et al., 2005); 0.13–1.7 (Belaid et al., 2019); 0.08 (de Paula et al., 2009); 0.08 (Mola et al., 2024, advanced)	5.0	Belaid et al., 2019; de Paula et al., 2009; Feki et al., 2025; Rattan et al., 2005, 2010; Mola et al., 2024
Mn	not reported	0.04–0.17 (Feki et al., 2025); 0.024–0.122 (Rattan et al., 2005); 0.015 (de Paula et al., 2009); 0.0571 (Macrì et al., 2024, effluent for reuse)	0.20	de Paula et al., 2009; Feki et al., 2025; Rattan et al., 2005, 2010; Macrì et al., 2024

Abbreviations: FAO, Food and Agriculture Organization of the United Nations; LOQ, limit of quantification; ND, not detected; WHO, World Health Organization.

## 3 TWW IMPACTS ON CRITICAL SOIL BIOCHEMICAL NITROGEN PROCESSES

TWW irrigation delivers water, easily mineralizable organic matter, nutrients, heavy metals, and various anthropogenic organic substances, such as antibiotics, ARGs, and microplastics, which may impair or reshape microbial communities and activity, changing the overall nitrogen dynamics in soil–water–plant–atmosphere system (Figure 1). In this section, a thorough review of the current knowledge on potential TWW effects on critical soil nitrogen processes, along with their critical drivers evidenced in soil receiving TWW, is carried out, emphasizing on fundamental processes, including mineralization, nitrification, ammonia volatilization, and denitrification.

**FIGURE 1:**
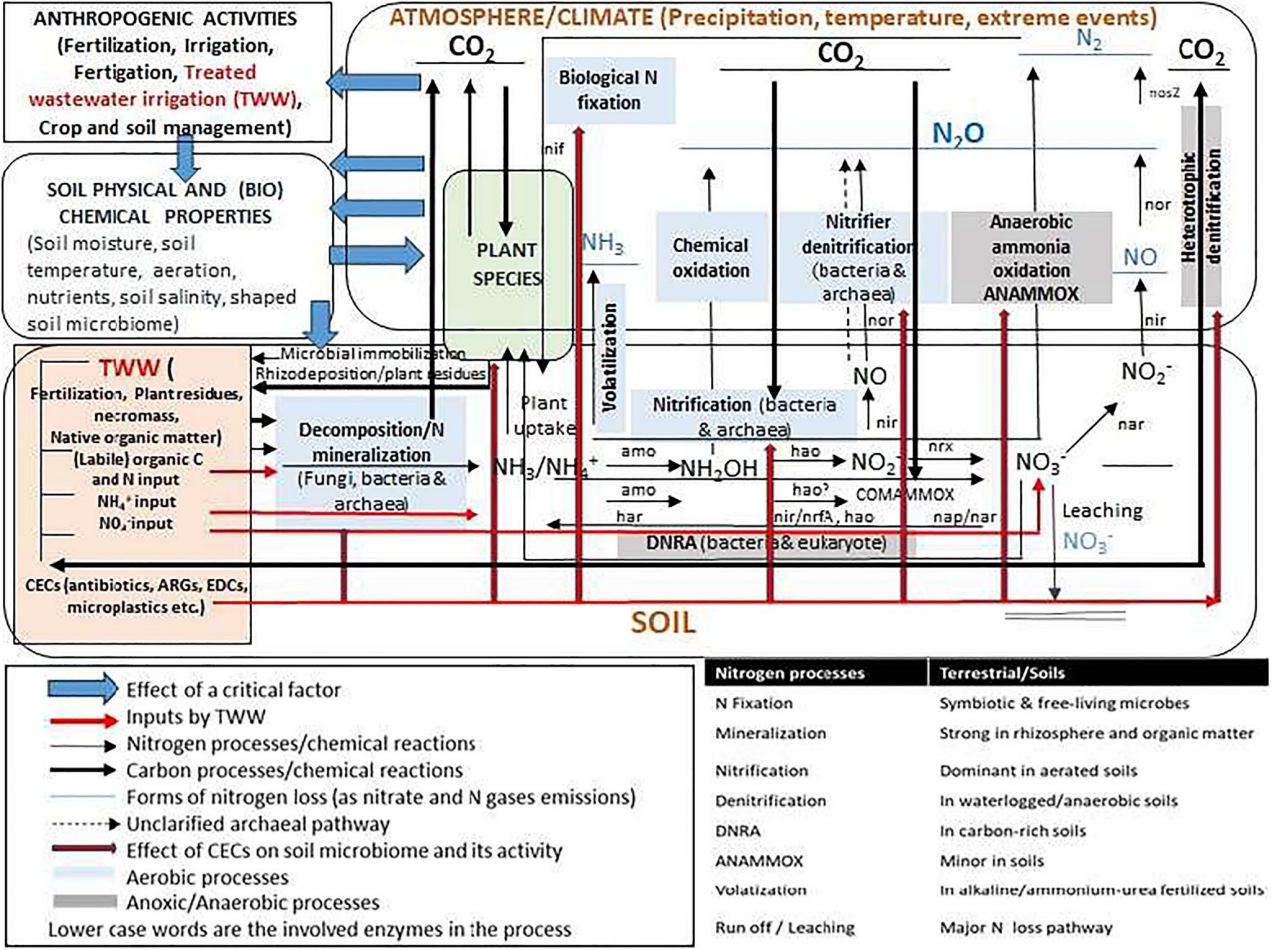
Diagram illustrating nitrogen and carbon turnover processes in treated wastewater (TWW)‐irrigated agroecosystems, with or without fertilization. TWW irrigation introduces water, labile organic carbon, nitrogen, and micropollutants (e.g., contaminants of emerging concern [CECs]) into the soil–plant–water–atmosphere, all co‐regulating critical nitrogen (and carbon) processes (decomposition, N mineralization, nitrification, autotrophic (nitrifier) and heterotrophic denitrification, dissimilatory nitrate reduction to ammonium (DNRA), anaerobic ammonia oxidation (ANNAMOX), and biological N fixation). Process‐specific enzymes and relevant encoding genes are also included in the diagram.

### 3.1 Microbial mediation and N transformations

#### 3.1.1 Soil microorganisms and enzymatic activity

Organic N mineralization is a critical turnover process in the terrestrial nitrogen cycle (Figure 1), determining its soil retention and supply to microorganisms and plants; it refers to the turnover of organic N to inorganic forms (i.e., ammonium or nitrate). Nitrogen mineralization refers to the depolymerization of organic compounds by extracellular enzymes excreted by heterotrophic microorganisms, including bacteria and eukaryotes (i.e., fungi). So far, bacterial heterotrophs isolated from various environments include members of the bacterial phyla Proteobacteria, Firmicutes, Acidobacteria, Actinobacteria, Bacteroidetes, Verrucomicrobia, Planctomycetes, Cyanobacteria, or fungal phyla such as Chytridiomycota, Blastocladiomycota, Neocallimastigomycota, Glomeromycota, Ascomycota, and Basidiomycota (Figure 2).

**FIGURE 2:**
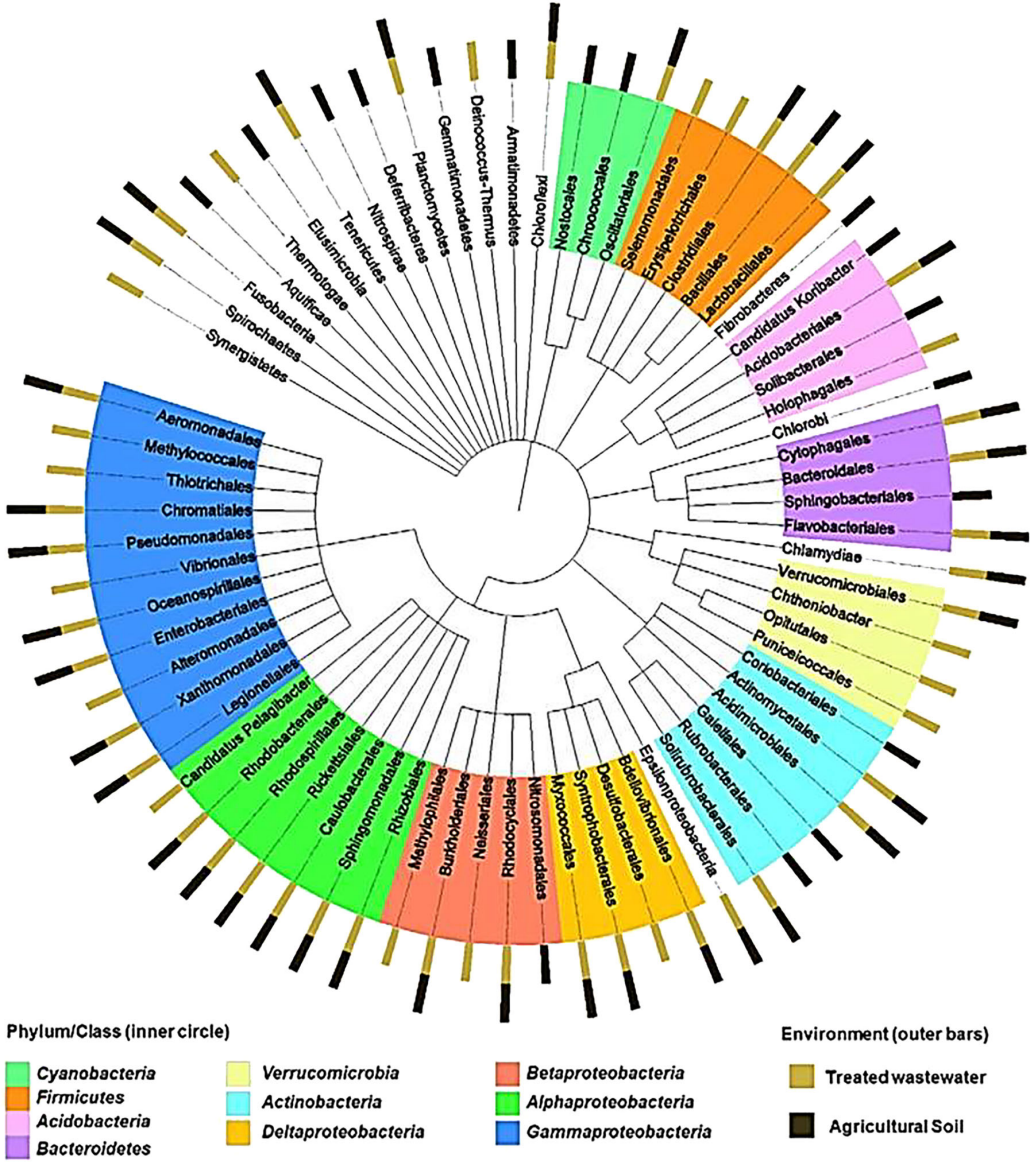
Dendrogram of bacterial diversity observed in treated wastewater (TWW) and agricultural soil (Becerra‐Castro et al., 2015).

Reviewed literature shows that TWW irrigation does not uniformly stimulate microbial biomass, community structure, or diversity. The direction and magnitude of the effect depend strongly on treatment level, water quality, irrigation duration, and the specific microbial parameter measured, a pattern that becomes clear only once studies are compared on these terms (Table 5). There is clear evidence that increasing treatment levels reduce microbial biomass; for example, soil bacterial biomass increased only under raw wastewater, but there was no significant change under constructed wetland or membrane‐bioreactor reclaimed water (RW) (Moulia et al., 2023). Similarly, raw or under‐treated sewage increased in biomass, but this inefficiency cannot be generalized to secondary and tertiary TWW (Lal et al., 2015; Rosin et al., 2020). However, a four‐level lysimeter gradient study showed microbial biomass C highest in secondary rather than tertiary treatments, and a separate paired comparison at another site did not show significant differentiation between TWW and freshwater irrigation (Heinze et al., 2014), both indicating that increasing treatment does not necessarily translate into microbial suppression and that site‐specific factors may co‐affect. An apparent TWW effect on microbial biomass disappeared once land‐use history was properly accounted for in one comparison: biomass increased significantly and equally in both irrigated and nonirrigated control plots over 2 years, driven by willow establishment rather than by TWW irrigation (Truu et al., 2009).

**TABLE 5:** Examples of treated wastewater (TWW) effects on soil microorganisms.

Driver	Parameter	Treatment level	Finding	Direction	Source
Long‐term (∼100 years) vs. never‐irrigated	Bacteria/actinomycetes/fungi counts	Not stated	Highest counts in organic particles of long‐term irrigated soil	↑	Filip et al., 2000
TWW (>20 years) vs. non‐TWW	Microbial biomass C	Secondary	Significantly higher (non‐paired, non‐contemporaneous comparison—weaker evidence than paired designs)	↑	Adrover et al., 2012
TWW vs. FW, paired (43 years)	AMF diversity	Secondary	Both lower under TWW (10 vs. 15 types; H′ 1.94 vs. 2.40)	↓	del Mar Alguacil et al., 2012
TWW vs. FW (Batsra)	Microbial biomass C	Secondary	FW higher (+12%), n.s.	↓ (n.s.)	Heinze et al., 2014
Lysimeter (four level)	Microbial biomass C	FW→UF→Tertiary→Secondary	Cmic highest under secondary TWW (229 µg/g, n.s.)—contradicts a simple “TWW suppresses biomass” narrative.	↑	Heinze et al., 2014
TWW vs. control	Microbial biomass (SIR)	Secondary	Increased equally in both irrigated and control plots over 2 years—confounded by willow establishment and baseline mismatch, not attributable to TWW	No TWW‐specific effect	Truu et al., 2009
TWW vs. potable	Bacterial composition	Advanced/Grade 1‐A	Proteobacteria significantly enriched under RW alone	Shift	Cui et al., 2025
TWW + sludge	Bacterial α‐diversity	Advanced/Grade 1‐A	Shannon higher in PW/RW vs. sludge treatments; diversity mainly sludge‐dose‐driven, not water‐quality‐driven	Sludge‐driven, not water‐driven	Cui et al., 2025
TWW + N fertilizer	Fungal/bacterial/actinomycete abundance	Tertiary	RW significantly increased all three groups (+21%–61%); fertilizer type had no significant effect.	↑	Chi et al., 2023
Advanced/tertiary vs. FW	Alpha diversity, composition, plant biomass	Advanced/tertiary	No significant alpha‐diversity change; TWW favored N‐cycling taxa; plant biomass significantly ↑ (taxonomic shift/plant growth, not demonstrated microbial biomass increase)	Mixed	Mola et al., 2024
Raw sewage vs. groundwater	Dehydrogenase, FDA, biomass C	Raw/untreated	All three significantly ↑ at 0–5 cm only; n.s. at 5–15 cm (values averaged over two confounded factors)	↑ (shallow only)	Rosin et al., 2020
Chronosequence (0→15 years)	Heterotrophic counts, biomass C, respiration	Not stated	All three significantly ↑, duration‐dependent (short conference paper, no full statistical detail)	↑	Hidri et al., 2018
Raw/under‐treated vs. GW	Biomass C, DHA/urease/phosphatase	Raw/under‐treated	MBC ∼1.3× higher; all three enzymes ↑; well‐controlled for fertilizer dose (no community/diversity data collected)	↑	Lal et al., 2015
Raw→secondary→advanced gradient	16S copy number, community, genes	Four‐level gradient	Biomass ↑ ONLY under raw water; no change under treated water—contradicts a general “TWW increases biomass” narrative; clean gradient design	↑ (raw only)	Moulia et al., 2023
Secondary chronosequence (0/1/7/15 years)	MAB, fungi, biomass C&N, DGGE	Secondary	MAB/fungi ↑ from Year 7; MBC/MBN ↑ from Year 1	↑ (time‐dependent)	Werheni Ammeri et al., 2023
40 years TWW pivots vs. control	Soil chemistry, diversity, N‐cycling taxa	Secondary→tertiary	Soil chemistry and prokaryotic diversity ↑; fungal diversity unaffected but composition shifted (treatment level spans a mixed 40‐year history, not a single clean stage)	Mixed	Chauhan et al., 2025
Secondary vs. GW, 7‐depth profile	Bacterial/fungal α/β‐diversity	Secondary	Bacterial α‐diversity ↑; fungal α‐diversity ↑ only at 50–100 cm; smallest β‐diversity divergence of three sources tested (domestic‐only values from a 3‐source study)	Mixed	Dang et al., 2019
3‐year forage rice, continuous TWW	pH, EC, water retention	Not stated	TWW ↑ pH stability, ↑ EC, ↑ water‐holding capacity (mechanism only; no biomass measured; cited alongside Mola et al., 2024, which reports the opposite direction for actual biomass—a 36% shoot‐biomass reduction under low‐N TWW)	Supports mechanism only	Sao et al., 2025
Secondary TSE, surplus irrigation	Functional/catabolic diversity (Biolog)	Secondary	Ss/H′ higher under TSE at most time points (culturable‐heterotroph catabolic diversity—not directly comparable to 16S/ITS/AMF metrics used elsewhere in this table)	↑	de Paula et al., 2009
Secondary vs. FW, 4 years orchard	FDA, dehydrogenase, respiration, nitrification	Secondary (upgraded mid‐study 2007)	FDA/DEH/respiration ↑ during irrigation season, returned to baseline each winter, effect NOT cumulative; nitrification result null, confounded by N‐matched FW control	↑ (seasonal only)	Elifantz et al., 2011

Abbreviations: AMF, arbuscular mycorrhizal fungi; DEH/DHA, dehydrogenase activity; DGGE, denaturing gradient gel electrophoresis; EC, electrical conductivity; FDA, fluorescein diacetate activity; FW, freshwater; GW, groundwater; ITS, internal transcribed spacer; MAB, mesophilic aerobic bacteria; MBC, microbial biomass carbon; MBN, microbial biomass nitrogen; n.s., not significant; PW, primary‐treated wastewater; RW, reclaimed water; SIR, substrate‐induced respiration; TSE, treated sewage effluent; UF, ultrafiltration.

Literature review on microbial diversity notably showed differentiation between bacterial and fungal communities within the same study more often than they agreed. For example, TWW irrigation with advanced/tertiary effluent did not have any significant change in bacterial and fungal alpha diversities; however, selective influence on microorganisms involved in nutrient cycling was demonstrated (Mola et al., 2024). In a 40‐year comparison, prokaryotic alpha diversity increased significantly under TWW in contrast to fungal, although the fungal community composition shifted markedly during the irrigation period (Chauhan et al., 2025). Also, a vertical soil‐profile study found a similar differentiation by soil depth, showing an increase in bacterial alpha diversity in the whole profile, while the fungal one was stimulated only at the deepest layer (50–100 cm) (Dang et al., 2019). By contrast, in a 43‐year study, paired comparison showed that both arbuscular mycorrhizal fungal sequence‐type richness and Shannon diversity indices were decreased under TWW than under freshwater irrigation (del Mar Alguacil et al., 2012), which was attributed to increased nutrient supply favoring fewer and more competitive microbial taxa. A further, distinct pattern (increase in richness and Shannon diversity under secondary‐treated sewage effluent than in nonirrigated soil) for functional (catabolic) diversity was also reported (de Paula et al., 2009).

Regarding the effect of TWW irrigation on microbial community composition and specific taxa, shifts have been recorded; however, the taxa affected and the true underlying driver varied and were not always attributable to TWW irrigation alone. RW alone enriched Proteobacteria relative to potable water; in the same study, however, bacterial Shannon diversity under combined TWW‐plus‐sludge treatments was found to be primarily sludge‐driven rather than driven by the irrigation water itself (Cui et al., 2025). In that study, TWW irrigation increased the abundance of *ermB* (erythromycin ribosome methylase gene B) and *sul1* (sulfonamide resistance genes (dihydropteroate synthase types 1 and 2) in rhizosphere soils (Cui et al., 2025). A domestic/leather‐industry/pharmaceutical wastewater comparison study showed a decrease in Actinobacteria and an increase in Planctomycetes under domestic TWW effluents; it was shown that TWW bacterial and fungal abundance responses were depth‐dependent rather than uniform with fungal operational taxonomic unit abundance decreasing at 0–30 cm and 50–100 cm layers but increasing at intermediate depth (30–50 cm) (Dang et al., 2019). A recent study suggests that the increase can be solely attributed to TWW irrigation: a wheat–maize rotation study reporting substantial increases in fungal, bacterial, and actinomycete abundance combining TWW irrigation with inorganic nitrogen fertilization (Chi et al., 2023). In addition to abundance, changes in the structure of soil microbiota were reported (Cui et al., 2025). Summarized presentations of certain TWW studies are provided in Table 5. Also, reviews on the mechanistic drivers underlying observed patterns involve several drivers, such as organic carbon and nutrient loading, TWW‐induced changes in soil pH, salinity, and various soil properties (e.g., water‐holding capacity) (Becerra‐Castro et al., 2015; Elifantz et al., 2011; Sao et al., 2025), all of which are addressed in detail in Section 3.2, together with the conditions that determine whether such effects persist over time or remain transient.

Enzymes implicated in biochemical processes involving nitrogen (and other elements) are sensitive indicators of changes occurring in soil under TWW irrigation. Observations on long‐ and short‐term field studies dealing with wastewater land application report more often increased enzyme activities relative to freshwater or nonirrigated controls (Brzezinska et al., 2006; Chatzakis et al., 2011; del Mar Alguacil et al., 2012; Filip et al., 2000) (Table 6). This stimulatory effect is neither uniform nor unconditional, strongly depending on the treatment level of RW, duration of irrigation, and factors that are rarely made explicit when different studies are compared. Across different sites in California spanning primary to tertiary treatment, overall soil enzyme activity increased 1.3‐ to 3.2‐fold relative to paired controls, with secondary‐treated sites showing the weakest and least consistent response (W. Chen, Wu, et al., 2008). A four‐level comparison between secondary, tertiary, and ultrafiltered wastewater over fresh water showed an increase in dehydrogenase activity in soil under secondary treatment (Heinze et al., 2014). In a recent 2‐year trial with comparisons between ultraviolet (UV) and ultrafiltration (UF)/UF‐treated effluents over water from a river showed increases in dehydrogenase activity and differences between treatments and soil depths were observed (Puškáčová et al., 2025). In a pot‐based comparison of three treatments irrigating urban shrubs, carbon‐ and phosphorus‐acquiring soil enzymes increased under water treated through anaerobic–anoxic–oxic processes, while nitrogen enzyme activity (N‐acetylglucosamines) increased under ecological or batch‐reactor‐treated effluents (Y. Zhou et al., 2025). Another important available evidence is the enzyme‐specific response to reclaimed wastewater irrigation. Thus, the observed discrepancies in the response of enzyme activity in TWW‐treated soils may indicate the co‐effects and interactions of the above important factors. A summary of the studies used for the enzyme section is provided in Table 6.

**TABLE 6:** Examples of treated wastewater (TWW) effects on soil enzyme activity.

Driver/comparison	Enzyme(s)	Treatment level	Finding	Direction	Source
TWW vs. no‐irrigation control	Dehydrogenase, urease, acid, and alkaline phosphatase	Secondary	Higher than no‐irrigation control, though lower than a freshwater‐irrigated control for all enzymes except urease under Salix	↑ (vs. no‐irrigation)	Brzezinska et al., 2006
TWW vs. N‐equalized freshwater	FDA hydrolysis, dehydrogenase	Secondary → upgraded secondary (paired, N‐equalized control)	Higher only during irrigation season; effect described as relatively minor over the longer term	↑ (seasonal only)	Elifantz et al., 2011
Long‐term (∼100 years) vs. never‐irrigated	β‐glucosidase, β‐acetylglucosaminidase, proteinase	Not stated	Higher, especially in organic soil fractions (three‐way paired design)	↑	Filip et al., 2000
Same site, irrigation stopped 20 years prior	Same enzymes	Same	Still elevated 20 years after irrigation stopped	↑ (attenuated, persistent)	Filip et al., 2000
Same study as above	β‐glucosidase, alkaline phosphatase	Secondary	Significantly higher (cropping pattern differed alongside irrigation, so cropping cannot be fully excluded as a contributing factor)	↑	Adrover et al., 2012
TWW vs. FW, paired, simultaneous (43 years)	Dehydrogenase, urease, protease, alkaline phosphatase, β‐glucosidase	Secondary	All five enzymes significantly higher (well‐controlled paired design)	↑ (all five)	del Mar Alguacil et al., 2012
TWW vs. paired control, 5 sites	Composite of 14 enzymes (cellulase, phosphatases, dehydrogenase, etc.)	Primary/secondary/tertiary (site‐dependent)	Overall activity higher (composite range 1.3–3.2×); secondary‐treatment sites showed the weakest response	↑ (heterogeneous by site)	W. Chen, Wu et al., 2008
TWW vs. FW (Batsra)	Enzyme diversity (Shannon–Weaver index)	Secondary	Significantly higher under TWW, notably in the opposite direction to the activity‐level result at the same site, illustrating that diversity and activity need not move together	↑	Heinze et al., 2014
TWW vs. FW (Gaza)	Amino‐peptidase	Secondary	Higher under FW, not significant	↓ (n.s.)	Heinze et al., 2014
Same study (previously unreported finding)	Alkaline phosphatase	Secondary	Significantly increased under TWW	↑	Truu et al., 2009
N addition (general, not TWW‐specific)	N‐acetyl‐glucosaminidase (NAG), urease, proteases, aminopeptidases	N/A—meta‐analysis, cited for mechanistic context only	NAG +5.5%, urease +11.6%; proteases flat or decreased	Enzyme‐specific, mixed	Chen et al., 2018
TWW (three levels) vs. river water, shallow (0–25 cm)	Dehydrogenase	Secondary/UV‐treated/UF‐UV‐treated	Steady‐state increase vs. river water: +20.5%/+18.2%/+23.4% across the three treatment levels	↑	Puškáčová et al., 2025
Same study, deep layer (25–45 cm)	Dehydrogenase	Same three levels	Steady‐state decrease vs. river water: −12.0%/−15.0%/−2.5%, opposite direction to the shallow layer at the same site	↓ (opposite of shallow layer)	Puškáčová et al., 2025
W1 (A2O‐treated) vs. tap water, urban pot trial	β‐cellobiosidase, β‐xylosidase	Secondary (A2O)	Significantly highest of all treatments tested, at both 3‐ and 5‐month sampling	↑ (W1 specifically)	Y. Zhou et al., 2025
W2/W3 vs. tap water and W1	N‐acetyl‐glucosaminidase	Secondary (BBR)/secondary + ecological polishing	Elevated at 3 months; no longer different by 5 months	↑ then no change	Y. Zhou et al., 2025
W1 (A2O‐treated) vs. tap water	Phosphatase	Secondary (A2O)	Consistently highest in W1 at both sampling times	↑ (W1 specifically)	Y. Zhou et al., 2025

Abbreviations: A2O, anaerobic–anoxic–oxic; FW, freshwater; N/A, not applicable; n.s., not significant; UF, ultrafiltration; UV, ultraviolet.

#### 3.1.2 Nitrification

Assessment of TWW impact on nitrification processes requires consideration of the abundance, diversity, and nitrification activity of the major groups of ammonia‐oxidizing prokaryotes. So far, it is known that nitrification, a key two‐step process comprising the oxidation of NH_4_^+^ to NO_2_^−^ and NO_3_^−^, is driven by discrete clades of beta‐ and gamma‐proteobacteria, that is, ammonia‐oxidizing bacteria (AOB), and ammonia‐oxidizing archaea (AOA), and nitrite‐oxidizing bacteria (NOB) (X. Lu et al., 2015; Martens‐Habbena et al., 2009; Spang et al., 2010; Treusch et al., 2005). Apart from AOA and AOB, members of the *Nitrospira* genus known as Comammox (complete ammonia oxidation) bacteria have been found to be capable of performing complete nitrification (ammonia to nitrate) (Daims et al., 2015) (Figure 1). A literature review on TWW irrigation does not show a uniform effect on nitrification or on the ammonia‐oxidizing communities, which highly depends on treatment level, nitrifier group, and duration of irrigation (Table 7); TWW irrigation was found to stimulate, inhibit, or leave nitrification unaffected, and importantly, a potential change in community composition does not necessarily translates into a change in nitrification activity. Thus, a comparison among raw activated‐sludge RW, groundwater, and primary‐treated effluent showed higher archaeal *amo*A (ammonia monooxygenase subunit A gene) abundance under properly treated (activated‐sludge) water than groundwater or primary‐treated effluent: the latter suggests a relatively high organic load, which paradoxically suppressed autotrophic nitrification, perhaps via an elevated C:N ratio (Chi et al., 2024). In a century‐scale raw‐wastewater chrono sequence, a fivefold increase in ammonia oxidizer abundance (based either on 16S rRNA [16S ribosomal RNA] gene relative abundance or *amo*A gene copies) and a shift in the AOA:AOB ratio from 9.8 to 1.6 were found, indicating more favorable conditions for AOB rather than AOA (Aguilar‐Rangel et al., 2024), but this study explicitly used untreated municipal wastewater.

**TABLE 7:** Examples of treated wastewater (TWW) effects on nitrifiers and nitrification.

Driver/comparison	Parameter	Treatment level	Finding	Direction	Source
Raw/untreated WW chronosequence (0/25/50/100 years)	*amo*A‐AOA/AOB, AOA:AOB ratio, community structure	Raw/untreated	AOA relative abundance (16S%) approximately doubled; AOB *amo*A copies increased ∼10‐fold by 100 years; AOA: AOB ratio shifted from 9.8 to 1.6; community structure shift driven by pH (AOB) and C: N ratio (AOA); Shannon diversity dipped at 25 years before partial recovery. Internally robust for this raw/untreated chronosequence but should not be generalized to secondary/tertiary‐treated TWW.	↑ (AOB favored over AOA)	Aguilar‐Rangel et al., 2024
Advanced/Grade 1‐A RW vs. PW ± sludge dose	AOA‐*amo*A, AOB‐*amo*A	Advanced/Grade 1‐A	AOA unaffected by any treatment tested; AOB increased only under reclaimed water combined with high‐dose sludge—reclaimed water alone was insufficient to drive the AOB effect.	No effect (AOA); ↑ (AOB, sludge‐dependent)	Cui et al., 2025
Tertiary RW vs. groundwater, 2 × 2 with fertilizer type	AOA‐*amo*A, AOB‐*amo*A, NOB	Tertiary	AOA increased under reclaimed water; AOB and NOB unaffected.	↑ (AOA only)	Chi et al., 2023
Secondary WW vs. control, willow coppice, 2 years	Potential nitrification	Secondary	No significant difference between irrigated and control plots.	No effect	Truu et al., 2009
RW vs. CW (deionized), 3 N‐forms × 2 rates, disturbed‐soil incubation	AOA‐*amo*A, AOB‐*amo*A, NOB; N_2_O emissions	Tertiary (same site as Chi et al., 2023)	Reclaimed water increased AOA and AOB gene abundance as a main effect, with no NOB effect, but the response was highly conditional on fertilizer type/rate and reversed in several sub‐conditions; N_2_O increased only under high‐NH_4_^+^ and NO_2_^−^ conditions, not NO_3_^−^. Reported changes reflect gene abundance and emissions rather than a direct measurement of nitrification activity.	↑ (main effect only, strongly conditional)	Chi et al., 2025
Primary‐treated effluent vs. FW, 2 plant species, pot trial	AOA/AOB *amo*A gene copies; potential nitrification rate (PNR)	Primary	*amo*A gene copies declined following effluent application, a differential decline between taxa producing the observed ratio shift, not a late increase; potential nitrification rate increased 155%–250% relative to freshwater, depending on plant species.	↓ populations; ↑ PNR	Tsiknia et al., 2013
Secondary/“semi‐treated” effluent vs. water/rain‐fed × harvest‐residue retention	Gross nitrification rate (^15^N pool dilution)	Secondary (source uses “secondary” and “semi‐treated” inconsistently)	Gross nitrification reached 3.1 mg N/kg/day only when effluent application was combined with harvest‐residue retention; without residues the rate was 0.51 mg N/kg/day, similar to water‐irrigated soil—the effect depends on a co‐applied carbon substrate rather than effluent alone.	↑ (conditional on residue co‐factor, not effluent alone)	Livesley et al., 2007
Secondary RE vs. FW, Grumosol, lysimeter + lab incubation (2 moisture levels)	Gross N nitrified (^15^N pool dilution)	Secondary	Nitrified nitrogen was 15%–25% lower under reclaimed effluent than freshwater, consistently at both field‐capacity and saturation; oxygen stress from organic‐matter oxidation confirmed as the mechanism.	↓ (both moisture levels)	Master et al., 2003
Secondary effluent vs. nutrient‐matched FAW, lysimeter + orchard	AOB community composition (*amo*A DGGE); NO_3_^−^/NH_4_^+^ function	Secondary	Nitrosomonas‐like taxa appeared alongside persisting Nitrosospira‐like taxa, an addition to, not a replacement of, the existing community, attributed to organic matter/BOD content, with ammonium, pH, and EC explicitly ruled out via a nutrient‐matched control; no functional (NO_3_^−^/NH_4_^+^) difference despite the compositional shift.	Shift (complexity ↑); function unchanged	Oved et al., 2001
TWW effluent vs. tap water, two soils (Arenosol/Cambisol), 152‐day season	AOB, AOA, Nitrospiraceae (comammox)	Not stated	AOB fully resilient (no effect in either soil or at any time point); AOA decreased only in the Cambisol, only early in the season; comammox Nitrospiraceae decreased only in the Cambisol, only at the final time point; resilience was group‐specific rather than uniform.	No effect (AOB); decreased, soil‐ and time‐specific (AOA, Nitrospiraceae)	Chroňáková et al., 2024
40‐year TWW pivots vs. control (secondary→tertiary, transitioned 2012)	Nitrosomonadaceae, Nitrosopumilaceae, Nitrososphaeraceae, Nitrosotaleaceae, Nitrospiraceae	Secondary→tertiary	Significant water‐quality effect on relative abundance of all ammonia‐oxidizer/comammox families tested, with summer‐specific stimulation of the AOA families Nitrososphaeraceae/Nitrosopumilaceae, reaching up to ∼20% of the total prokaryotic community.	↑ (AOA, summer‐specific)	Chauhan et al., 2025
RW (activated sludge) vs. UW vs. PW (primary), 2‐year field + incubation	AOA‐*amo*A, AOB‐*amo*A, *nir*K, *nir*S, *nos*Z; N_2_O source partitioning	Three‐level: primary/secondary‐equivalent/groundwater	AOA‐*amo*A increased under RW vs. both UW and PW; AOB‐*amo*A unaffected; *nir*K/*nir* S increased vs. UW only, not vs. PW; *nos*Z unaffected by any treatment; autotrophic nitrification was the dominant N_2_O pathway across all treatments (60%–72%), highest under RW (71.77%); PW's higher organic load paradoxically suppressed autotrophic nitrification via an elevated C: N ratio.	↑ (AOA, *nir*K/*nir*S; treatment‐level dependent)	Chi et al., 2024

Abbreviations: AOA, ammonia‐oxidizing archaea; AOB, ammonia‐oxidizing bacteria; BOD, biochemical oxygen demand; CW, control water (deionized water); DGGE, denaturing gradient gel electrophoresis; EC, electrical conductivity; FW, freshwater; N/A, not applicable; NOB, nitrite‐oxidizing bacteria; PW, primary‐treated wastewater; RE, reclaimed effluent; RW, reclaimed water; UW, underground water; WW, wastewater.

Different responses between AOA and AOB to TWW irrigation have been recorded, but the direction of that differentiation is not consistent across studies. For example, in tertiary/advanced‐treated TWW irrigation, increased AOA's *amo*A gene copies but did not significantly affect the abundance of AOB and NOB genes in the soil (Chi et al., 2023). In a microcosm study, TWW irrigation increased the abundances of AOA‐*amo*A and AOB‐*amo*A but did not affect NOB abundance (Chi et al., 2025); a disturbed‐soil incubation further complicates this, as the apparent pattern reported as a main effect was in fact strongly conditional on fertilizer type and application rate, reversing or disappearing entirely in several sub‐conditions (Chi et al., 2025). In advanced/Grade 1‐A RW versus primary‐treated wastewater (PW) ± sludge dose experiment, AOB gene abundance responded positively to TWW irrigation combined with high sludge doses, while AOA remained unaffected by TWW irrigation (Cui et al., 2025). Yet in a temperate two‐soil field trial, the opposite pattern held: AOB (Nitrosomonadaceae) were fully resilient to TWW irrigation across both soils and the entire growing season, while AOA and comammox Nitrospiraceae showed soil‐ and time‐specific declines (Chroňáková et al., 2024). Taken together, nitrifier tolerance and sensitivity should be a subject of group‐specific properties and not solely dependent on inherent attributes of AOA or AOB.

A well‐controlled lysimeter and orchard comparison, using a fertilizer‐amended control water matched to the effluent's nitrogen content, found that TWW irrigation added Nitrosomonas‐like AOB populations alongside the *Nitrosospira*‐like populations that persisted from the freshwater‐irrigated soil, an increase in community complexity, not a replacement of one dominant taxon by another (Oved et al., 2001). This shift was attributed to organic matter/biochemical oxygen demand rather than to ammonium, pH, or EC, all of which were explicitly ruled out by the matched‐nutrient design (Oved et al., 2001). A 40‐year comparison similarly confirmed significant water‐quality effects on the relative abundance of ammonia‐oxidizing and comammox families, with archaeal taxa (Nitrososphaeraceae and Nitrosopumilaceae) specifically stimulated in summer to nearly 20% of the total prokaryotic community (Chauhan et al., 2025). This general principle, which claims that functional redundancy allows nitrification activity to be preserved even as the underlying community composition changes, is consistent with a broader synthesis of the literature; it should be noted that genetic diversity within the ammonia‐oxidizer has been observed to decline after long‐term (20‐year) irrigation even where function is maintained (Lopes et al., 2016).

The literature discussed above indicates stimulation of nitrifier abundance or gene copy number under TWW irrigation. However, at least one well‐controlled study provides opposite evidence: reclaimed effluent application inhibited gross nitrification by 15%–25% relative to freshwater irrigation and inhibition that persisted at both field capacity and saturated soil moisture conditions. Authors claimed that O_2_ consumption from the oxidation of organic compounds in the effluent was responsible (Master et al., 2003). A decrease or late increase in AOA and AOB populations was reported by mesocosm studies planted with specific species under PW effluent (Tsiknia et al., 2013). The authors of that study claimed that the observed nitrifiers’ response was due to antagonism with heterotrophs for ammonia, without, however, excluding potential nitrifiers’ inhibition by wastewater composition. On the other hand, the study reported greater rates of potential nitrification in pots treated with wastewater than in control pots, from 155% to 250%, depending on planted species, suggesting adaptation of nitrifier communities due to changing soil conditions. The above indicates that organic loading can plausibly suppress nitrification by stimulating the abundance of soil microorganisms that can consume O_2_ and/or outcompete for available ammonia in the soil.

Several studies illustrate that factors other than TWW quality may influence nitrification response. For example, a willow‐coppice trial showed no significant difference in potential nitrification between secondary‐treated wastewater and untreated control plots (Truu et al., 2009); finally, a harvest‐residue/irrigation factorial trial found an increase in gross nitrification rate due to the combined effluent irrigation and harvest‐residue retention (Livesley et al., 2007). Changes in the community structure of both AOA and AOB after long‐term wastewater irrigation were reported, related mainly to changes in soil pH (particularly concerning AOB) and in the content of carbon and nitrogen (for AOA communities) (Aguilar‐Rangel et al., 2024).

#### 3.1.3 Denitrification and ammonia volatilization

Agricultural and natural soils are major global sources of N_2_O emissions, varying from 4.3 to 5.8 and 6 to 7 Tg_N_/year, respectively (Syakila & Kroeze, 2011), mostly related to soil microbial activity (Butterbach‐Bahl et al., 2013). The N_2_O emissions are mainly attributed to heterotrophic denitrification; however, this was challenged due to the contribution of nitrifying bacteria and archaea through nitrifier denitrification or by incomplete oxidation of hydroxylamine (NH_2_OH) to NO and N_2_O (Arp & Stein, 2003; Caranto et al., 2016). Recent studies demonstrate that archaea have lower N_2_O yields compared to bacterial ammonia oxidizers (Hink et al., 2016, 2018; Tzanakakis et al., 2019); the mechanisms of N_2_O production by archaea, as well as the nitric oxide reductase gene involved, have not been fully elucidated (Blainey et al., 2011; Kozlowski et al., 2016; Stieglmeier et al., 2014; Walker et al., 2010).

Denitrification is an important pathway of gaseous N losses from TWW‐irrigated soils, caused by the presence of organic carbon and nitrogen in effluents and of denitrifying microorganisms. TWW with or without N fertilization augmentation can substantially affect N_2_O emission from fields (Table 8). In a wheat–maize rotation field study in the presence of inorganic N fertilization, TWW irrigation increased concentrations of N_2_O by 12.33%–73.82%, NO by 13.74%–36.59% within a depth range of 0–30 cm, and of NO_2_^−^ after fertilization, accompanied by an increase in AOA (as *amo*A gene copies). Moreover, TWW irrigation increased the abundance of *nir*K (copper‐containing nitrite reductase gene), *nir*S (cytochrome cd₁ nitrite reductase gene), and *nos*Z (Nitrous oxide reductase gene) denitrifying genes (Chi et al., 2023), in agreement with the findings of an earlier study with untreated wastewater, particularly concerning the *nir*K gene (Z.‐F. Zhou et al., 2011). Increases in N_2_O were reported due to TWW irrigation and nitrogen fertilization in a pot study (Tao et al., 2024). In line with this, TWW irrigation significantly increased N_2_O by 33.8% relative to control when no fertilizer N was present, but only by 4.1% once urea fertilizer was already applied (source × N‐fertilizer interaction significant). This suggests that TWW irrigation has a marginal contribution to N_2_O in fertilized soils, also showing an increasing trend with increasing soil moisture (%WFPS [water‐filled pore space]) (Xue et al., 2012). Similarly, in a field TWW irrigation study, based on ^15^N isotope labeling, the contribution of nitrification to N_2_O was 60.46%, observed in comparison with groundwater irrigation. The study showed parallel autotrophic nitrification as another important denitrifying pathway; N_2_O production strongly correlated with AOA‐*amo*A, *nir*S, and *nir*K (Chi et al., 2024). In a long‐term (4‐year) irrigation study with reclaimed wastewater (with or without N fertilization), a small but insignificant increasing trend in overall GHG emissions was observed (Chi et al., 2020). In microcosm studies, stimulation of N_2_O emissions by TWW addition with a cyclic increase‐and‐reduction pattern was reported (Tao et al., 2022). Recent studies reported no N_2_O emissions change when nitrate‐based fertilizers are applied; however, they showed an increase following ammonium‐based fertilization, accompanied by a minimal increase in *nir*K and *nos*Z gene copies. This highlights the contribution of nitrification to N_2_O emissions, supported by the greater abundance of AOA‐*amo*A and AOB‐*amo*A (Chi et al., 2025). Also, nitrification and denitrification appeared equally important in a field/lysimeter component study (Master et al., 2003). Recently, TWW irrigation showed no significant effect on AOA‐*amo*A, AOB‐*amo*A, *nir*K, *nir*S, and *nos*Z (Cui et al., 2025).

**TABLE 8:** Examples of treated wastewater (TWW) effects on denitrification.

Driver	Parameter	Treatment level	Finding	Direction	Source
Tertiary RW vs. UW, wheat‐maize rotation ± inorganic N fert.	N_2_O, NO concentrations (0–30 cm); *nir*K, *nir*S, *nos*Z	Tertiary	N_2_O +12.33%–73.82%; NO +13.74%–36.59%; *nir*K/*nir*S/*nos*Z all ↑	↑ (all)	Chi et al., 2023
RW (activated sludge) vs. UW vs. PW, ^15^N isotope field study	N_2_O pathway partitioning; correlation with AOA‐*amo*A, *nir*S, *nir*K	Three‐level (secondary‐equiv/primary/groundwater)	Autotrophic nitrification contributed 60.46% of N_2_O under RW (a contribution share, not an emission increase); N_2_O strongly correlated with AOA‐*amo*A, *nir*S, *nir*K	↑ Nitrification‐pathway share	Chi et al., 2024
RW ± N fert., 4‐year long‐term field study	Overall GHG emissions	Not stated	Emissions “slowly increased… not significant” per source's own wording—a small real trend, not zero effect	↑ (small, n.s.)	Chi et al., 2020
RW + high‐dose sludge vs. RW alone vs. PW	AOA‐*amo*A, AOB‐*amo*A, *nir*K, *nir*S, *nos*Z	Advanced/Grade 1‐A	RW alone: no effect on any of these genes. RW + high‐dose sludge: AOB significantly ↑	No effect (RW alone); ↑ AOB (RW + sludge)	Cui et al., 2025
Secondary RE vs. FW, Grumosol, lysimeter + lab (2 moisture levels)	N_2_O production; nitrification vs. denitrification contribution	Secondary	“Equally important” (54%/50%) confirmed for the field/lysimeter component only; lab incubation under saturation showed denitrification dominant (∼75%)	No effect (N_2_O); ∼equal share (field only)	Master et al., 2003
Long‐term TWW pivots (secondary→tertiary) vs. control	*nir*S, *nos*Z community structure	Secondary→tertiary	Significant water‐quality × season effect on *nir*S/*nos*Z structure	Shift (quality‐ and season‐dependent)	Chauhan et al., 2025
CW vs. RW vs. WW (raw), Beijing, qPCR + DGGE	*nir*K, *nir*S, *nos*Z abundance and structure	RW = treated; WW = Raw/untreated	*nir*K ↑ under both RW and WW; *nir*S ↑ only under WW, not RW; *nos*Z unaffected by either; *nir*S/*nir*K structure changed, *nos*Z did not	↑ *nir*K (both); ↑ *nir*S (WW only); no effect (*nos*Z)	Z.‐F. Zhou et al., 2011
GW vs. RW vs. LW ± nitrification/urease inhibitors, pot experiment, two cycles	*nar*G, *nir*S, *nos*Z; N_2_O emissions; NH_4_^+^/NO_3_^−^	Not stated	RW/LW irrigation increased N_2_O via NH_4_^+^‐N, NO_3_^−^‐N and gene abundance (confirmed, but a secondary finding, not the paper's main focus; main focus is inhibitor efficacy); *nar*G decreased with inhibitors; *nxr*A + *nar*G were the key SEM regulators	↑ (RW/LW baseline)	Tao et al., 2024
TWW vs. distilled water (CK), silt loam, incubation, 4 WFPS × ± urea factorial	Cumulative N_2_O emissions	Not stated	TWW ↑ N_2_O overall; +33.8% without N fertilizer (101.79 vs. 76.10 µg N/kg) but only +4.1% with urea already present (122.15 vs. 117.32 µg N/kg), S×N interaction significant, *p* < 0.01; TWW + urea combined was also sub‐additive vs. the simple sum of individual effects (*p* < 0.05)	↑ (strong, unfertilized); ↑ (weak, fertilized)	Xue et al., 2012

Abbreviations: AOA, ammonia‐oxidizing archaea; AOB, ammonia‐oxidizing bacteria; CW, control water (deionized water); DGGE, denaturing gradient gel electrophoresis; FW, freshwater; GHG, greenhouse gas; GW, groundwater; LW, livestock wastewater; n.s., not significant; PW, primary‐treated wastewater; qPCR, quantitative polymerase chain reaction; RE, reclaimed effluent; RW, reclaimed water; SEM, structural equation modeling; UW, underground water; WFPS, water‐filled pore space; WW, wastewater.

Considering all these outcomes, it can be concluded that TWW irrigation can increase soil N_2_O emissions and stimulate several denitrification‐related genes (*nir*K, *nir*S, and *nar*G [membrane‐bound nitrate reductase (α subunit) gene]), but the magnitude and even direction of these effects depend strongly on treatment level, co‐applied fertilizer, soil conditions, and which specific gene or process is examined. Long‐term TWW irrigation induced changes in community structures, evidenced by *nir*S and *nos*Z genes, depending on wastewater quality and seasonality (Chauhan et al., 2025); it was also seen to cause community structure changes in *nir*S and *nir*K genes, but not in *nos*Z genes (Z.‐F. Zhou et al., 2011).

Ammonia in TWW, with or without urea addition, and according to soil pH, may partially volatilize (in alkaline soils), be subject to microbial or plant uptake, or be oxidized. Earlier lysimeter/microcosm studies showed enhanced losses of NH_3_ after reclaimed effluent application due to NH_4_^+^ supply (Master et al., 2003). On the other hand, TWW irrigation significantly decreased cumulative NH_3_ emissions by 35% without nitrogen fertilizer application in paddy soils but caused an increase in NH_3_ emissions when applied in combination with nitrogen fertilization, with the former being attributed to improved yield (M.‐Y. Li, Wang, et al., 2020). This suggests that besides soil pH, NH_3_ volatilization can also be regulated by the co‐effect of other factors, such as NH_4_^+^ microbial assimilation and consumption (e.g., ammonia oxidation) and plant uptake.

#### 3.1.4 Inorganic N forms

Inorganic nitrogen accumulation in soil as NO_3_^−^, NH_4_^+^, and NO_2_^−^ under TWW irrigation represents microbial activity, varying according to the RW quality‐treatment level, N loading, and experimental conditions (Table 9). Nitrate and ammonium typically move independently (Chi et al., 2024; Livesley et al., 2007); a four‐arm ozonation experiment showed that the two ions moved in opposite directions in three of four treatments (Junejo et al., in press). Water‐source quality (N loading), affected by the level of treatment, is an important factor for nitrate formation (Saleh et al., 2025). Raw/untreated wastewater accumulated nitrate, which followed a different depth pattern than treated effluent (Balkhi et al., 2023). Plant species and soil type can outweigh water quality entirely: primary treated effluent on two species resulted in divergent nitrate and ammonium accumulation patterns (Tsiknia et al., 2013). Also, a two‐soil experiment showed that ammonium and nitrate were driven by soil type (Cambisol > Arenosol), and the nitrate effect concentrated almost entirely in one soil (Chroňáková et al., 2024). Nitrite is rarely measured in TWW‐irrigated soils; however, a case of effluent application generated marked nitrite build‐up (up to 9 mg N/kg short‐term and 30 mg N/kg longer term), suggesting the potential of decoupling between nitrification steps (Master et al., 2003). This effect may be related to treatment level (Chi et al., 2025); however, treatment level and aeration do not scale linearly with nitrate (Chi et al., 2024). Also, un‐ozonated RW accumulated more nitrate than the ozonated version of the same water despite added dissolved O_2_ (Junejo et al., in press). Finally, in a managed‐aquifer‐recharge soil‐column system, TWW addition increased ammonium depending on the anoxic wetting and drying, where nitrification was dominant (Silver et al., 2018).

**TABLE 9:** Examples of treated wastewater (TWW) effects on accumulation of inorganic N forms in soil.

Treatment level	NO_3_^−^	NH_4_^+^	Notes	Source
Secondary vs. nutrient‐matched FAW	Both treatments increased over the season at 15 cm; FAW was actually higher at final sampling (37 vs. 16 µg/g, no‐plant lysimeters)	Both increased then decreased, converging to near‐identical values by final sampling (0–10 µg/g)	Nutrient‐matched control design isolates the organic‐matter effect from the N‐supply effect—no TWW‐attributable difference detected in either ion.	Oved et al., 2001
Secondary (Grumosol)	Soil baseline 38.1 mg NO_3_^−^‐N/kg (field)/6.0 (lab)	Soil baseline 58.3 mg NH_4_‐N/kg (field)/12.0 (lab)	NO_2_^−^ increased distinctly under reclaimed effluent, especially at field capacity, up to 9 mg NO_2_^−^‐N/kg (30 in longer incubations)—accumulation linked to elevated pH/free‐NH_3_ conditions	Master et al., 2003
Secondary/“semi‐treated”	Soil water generally <10 mg/L (30 cm), <5 mg/L (100 cm) at normal rate; stored soil NO_3_^−^ not significantly increased vs. water irrigation	Soil water NH_4_^+^ higher than NO_3_^−^ at 100 cm—the reverse of the usual leaching‐risk assumption	Direct effluent NH_4_^+^ input plus indirect ammonification of soil organic N both implicated; NH_4_^+^, not NO_3_^−^, dominated downward N movement under TWW	Livesley et al., 2007
Tertiary RW vs. soil‐CW vs. soil‐RW	Water: RW 13.64 ± 4.12 mg/kg; Soil‐CW 9.12 ± 0.74; Soil‐RW 13.23 ± 0.37 mg/kg	Water: RW 7.21 ± 3.62; Soil‐CW 1.21 ± 0.12; Soil‐RW 0.81 ± 0.25 mg/kg (NO_2_^−^: 4.2 ± 0.42/0.56 ± 0.02/0.85 ± 0.14)	Soil‐RW shows lower NH_4_^+^ than soil‐CW despite the irrigation water itself containing more NH_4_^+^—water‐column N content does not translate directly into soil N content	Chi et al., 2025 (nitrification)
Three‐level: RW (activated sludge)/UW/PW (primary)	RW significantly higher than UW (SEM structural path, *p* < 0.05)	No significant RW effect vs. UW	Structural equation modeling identifies NO_3_^−^—not NH_4_^+^, TN, or SOC—as the specific pathway by which RW drives soil N_2_O emissions.	Chi et al., 2024
Primary	Soil‐solution NO_3_^−^ decreased in A. donax pots from late June onward vs. *E. camaldulensis* (*p* < 0.01)	Soil‐solution NH_4_^+^ higher in A. donax pots than in *E. camaldulensis* (*p* < 0.001, most samplings)	Same effluent applied to both plant species—the divergence is plant‐driven, not water‐quality‐driven	Tsiknia et al., 2013
Not stated	Significantly higher overall in effluent‐irrigated soils (*p* < 0.001), driven mainly by the Arenosol, not the Cambisol	No significant treatment effect; concentration pattern was soil‐type‐driven (Cambisol > Arenosol) rather than treatment‐driven	Soil type outweighs TWW as the driver of NH_4_^+^ concentration in this study	Chroňáková et al., 2024
A/O secondary RW ± O_3_^−^nanobubbles (4 arms)	Unozonated RW showed the highest NO_3_^−^ of all four treatments (*p* < 0.0001); ozonated arms also increased vs. control but less than plain RW	Mostly the opposite trend to NO_3_^−^ across the ozonated arms; only the ozonated‐tap‐water arm raised both ions simultaneously	Ozonation (added dissolved O_2_) did not straightforwardly increase soil nitrate beyond what unozonated RW already achieved.	Junejo et al., in press
2 secondary TWW sources (SBR vs. MLE), 2 sites × 2 fields × 2 years	One source: 6.8–9.7 → 23.6–24.8 mg/L (significant, *p* = 0.016–0.034); other source: 9.6–9.7 → 13.8–14.0 mg/L (not significant); higher at depth than surface	Significant at all four fields (*p* = 0.008–0.034), also higher at depth than surface	NH_4_^+^ response was consistent across both TWW sources; the NO_3_^−^ response depended on which treatment plant supplied the water—the lower quality Mahdasht (MLE) source drove the only significant NO_3_^−^ increase	Saleh et al., 2025
Raw/untreated (screening + grit removal only, explicitly stated by the source)	Increased at all depths, with the largest accumulation in the deepest layers (75–100 cm)—high mobility, substantial aquifer leaching risk	Increased only in the 0–75 cm layers; declined in the deepest layer (75–100 cm)—adsorbed, limited mobility.	Field N leaching (400 kg/ha) far exceeded crop uptake (185 kg/ha) due to severe over‐irrigation (efficiency only 29.5%)—a management artifact rather than an inherent TWW property. Fourth confirmed case of raw/untreated water in this review's source set (after Aguilar‐Rangel, Lal, Rosin), but the only one where the source states this openly rather than requiring detection	Balkhi et al., 2023
Not stated	Not reported	Slightly increased across the entire growth period under reclaimed water; peak concentration dose‐dependent (RW > 50%RW > GW), concentrated at 0–40 cm, negligible below 40 cm.	NH_4_^+^ shows a clear dose‐response to the proportion of reclaimed water in the irrigation blend, not just its presence/absence. The depth‐limited accumulation (cutoff ∼40 cm) separately suggests an adsorptive/reactive retention process rather than free downward leaching, consistent with the pattern also seen in Livesley et al., 2007	S. Lu et al., 2016

Abbreviations: CW, control water (deionized water); FAW, fertilizer‐amended water; GW, groundwater; MLE, modified Ludzack–Ettinger; PW, primary‐treated wastewater; RW, reclaimed water; SBR, sequencing batch reactor; SOC, soil organic carbon; TN, total nitrogen; UW, underground water.

### 3.2 Potential drivers of N transformations occurring in TWW‐irrigated soils

#### 3.2.1 Organic carbon and nitrogen

TWW irrigation, combined or not with organic and inorganic N fertilization, could increase carbon and nitrogen soil loading. In situations of non‐limiting nitrogen conditions and the presence of readily available carbon, nitrogen mineralization may be favored as organic nitrogen is used as a source, stimulating soil microorganisms. The influence of TWW irrigation on soil organic matter (SOM), dissolved organic carbon (DOC), and nitrogen concentration is not uniform and depends on irrigation duration, reclaimed wastewater treatment level, soil type, and the specific parameter measured (Table 9). For example, PW effluent caused a substantial increase in SOM after a short period (3 years) of irrigation (Tzanakakis et al., 2011). Secondary effluent later upgraded to tertiary, organic matter (OM) and TN accumulated significantly over 40 years of irrigation (Chauhan et al., 2025). Similarly, secondary‐treated effluent increased SOM from 0.1%–0.44% (control) to 0.23%–1.77% and 0.2%–1.93% after 27 and 16 years, respectively (Feki et al., 2025). Also, in a separate secondary chronosequence, total organic carbon, TN, and OM all increased significantly after 7 and 15 years (not 1 year) at depths down to 60 cm, alongside a parallel CEC increase, showing accumulation was not surface‐confined (Werheni Ammeri et al., 2023). Over 20 years of secondary (activated‐sludge) irrigation, SOM was statistically unchanged, but DOC was significantly elevated, which was attributed to dissolved organic matter in the wastewater itself and to alfalfa cropping (Adrover et al., 2012). In another study with secondary TWW, DOC was consistently higher than a nitrogen‐matched freshwater control for the first 2 years of a 4‐year trial, but this difference nearly disappeared after a treatment upgrade, showing the DOC effect is itself treatment‐level‐dependent (Elifantz et al., 2011); secondary and industrial‐treated effluent significantly elevated pore‐water DOC (Zolti et al., 2019). Over a single growing season under WWTP effluent (treatment level not stated), TWW unexpectedly decreased oxidizable organic carbon significantly in a Cambisol, probably via microbial priming of existing SOM; DOC also decreased (mainly in the Arenosol) while NO_3_^−^ increased (also mainly in the Arenosol) (Chroňáková et al., 2024); the authors noted that this short‐term decrease contrasts with long‐term TWW studies reporting SOM increases, suggesting duration determines the direction of the effect.

Consistent with the loading‐dependent framework, the microbial biomass and enzyme‐activity increases reported under raw or under‐treated sewage (Lal et al., 2015; Rosin et al., 2020); however, this was not evident under properly treated water within the same comparative design (Moulia et al., 2023), indicating the effect of organic and nutrient loading itself (Becerra‐Castro et al., 2015) and perhaps other factors discussed in the previous section above. It has been shown that autochthonous microbial biomass might be suppressed or stimulated, inducing changes in its diversity and community composition in various soil types, depending on native soil microbiome and the amount of added nitrogen (Cusack et al., 2011; Jangid et al., 2008; Jian et al., 2016; Liang et al., 2011; C. Wang, Lu, et al., 2018) (Table 1); Controversial responses of soil microbiome to nitrogen addition have been observed in various ecosystems, mainly correlated to soil microbial composition (Jian et al., 2016). For example, fungal populations tend to decline after heavier and longer nitrogen additions (Treseder, 2008; Wallenstein et al., 2006), although this is not always the case (Kamble & Baath, 2018; Treseder, 2008; C. Wang, Lu, et al., 2018), while bacteria are more resistant and may display group‐specific responses (Ramirez et al., 2012; C. Wang, Lu, et al., 2018). Regarding nitrifiers, variation in ammonia concentrations is possible in TWW‐irrigated soils, as mentioned above, affecting their population, community, and activity. This effect can be related to the inherent response of nitrifiers: AOA are characterized by a higher affinity than AOB (Prosser & Nicol, 2012), meaning that the former may dominate under low ammonia conditions, while the opposite may occur under high ammonia (Di et al., 2009; Jia & Conrad, 2009, 2010; Tzanakakis et al., 2018, 2022). Moreover, archaeal strains have been observed to perform alternative heterotrophic or mixotrophic metabolism (X. P. Chen, Zhu, et al., 2008; Jia & Conrad, 2009; Levicnik‐Hofferle et al., 2012; Offre et al., 2014; Tourna et al., 2011; Xu et al., 2012) relying on nitrifier‐specific genetics (Arp et al., 2007; Kim et al., 2011; Blainey et al., 2011). Taken together, TWW‐derived nitrogen and organic carbon (with or without added fertilization) may affect soil microorganisms, impacting their populations, community composition and structure, and the overall activity reflecting loading and microbial‐specific niches (e.g., discriminating ammonia oxidizers and affecting ammonia oxidation, plausibly explaining, at least in part, why organic‐load‐driven nitrification inhibition is reported in only some of the TWW studies reviewed here, rather than reflecting an independent source of unpredictability).

#### 3.2.2 Soil pH

TWW irrigation is associated with soil pH change across treatment levels, but direction varies with duration and site rather than following treatment category (Table 9). Secondary TWW increased soil pH (<1 unit) after short‐ or long‐term TWW irrigation (Adrover et al., 2012; de Paula et al., 2009; Feki et al., 2025) and, at the same calcareous site over a shorter 16‐year history, decreased by 0.54 units instead (Feki et al., 2025); a separate long‐term secondary‐effluent application shows pH declining with duration (Werheni Ammeri et al., 2023). However, the opposite trend is along an untreated wastewater, where pH increased over 25–100 years of irrigation (Aguilar‐Rangel et al., 2024). Tertiary‐treated effluents caused an increase in soil pH across different plant–soil combinations (Zolti et al., 2019), and it was significantly higher after 40 years of secondary/tertiary TWW irrigation (Chauhan et al., 2025). Advanced and high‐rate aerobic treatment produced a small pH decrease (J. F. Wang et al., 2007). Where nitrogen fertilization varied alongside water source, RW decreased pH relative to clean water at matched N rates, while reduced fertilization independently increased pH under either water source (W. Guo et al., 2018). In a Mediterranean land‐treatment system, an effect appeared only after the third of three application periods and only in the upper 15 cm (Tzanakakis et al., 2011). Studies found no pH effect: for example, in a nutrient‐matched experiment (Oved et al., 2001) and a secondary‐treated effluent irrigation in a willow plantation (Truu et al., 2009). Also, continuous TWW irrigation showed stable and near‐neutral soil pH, an effect attributed to organic acids and bicarbonate anions present in the wastewater (Sao et al., 2025).

Where a mechanism is proposed, it is consistently geochemical rather than nitrogen‐cycle mediated. Cation exchange accounts for most reported increases: TWW‐derived Ca and Na displace exchangeable cations at sorption sites, raising pH as these accumulate over decades (Adrover et al., 2012; de Paula et al., 2009; Feki et al., 2025). The same chemistry can reverse with less accumulated carbonate; salt‐driven Ca substitution in the argillo–humic complex produced a decrease instead at a 16‐year history of the same site (Feki et al., 2025). A distinct pathway involves effluent‐borne CO_2_/organic acids reaching equilibrium with soil CaCO_3_. No mechanism is proposed for several other reported shifts (Aguilar‐Rangel et al., 2024; Chauhan et al., 2025; W. Guo et al., 2018; Werheni Ammeri et al., 2023) reporting cation exchange and mechanism concerns about timing/extent, such as high buffering capacity delaying and spatially restricting the response to TWW addition (Tzanakakis et al., 2011).

Soil pH is known as the principal determinant of soil bacterial and fungal communities, exerting a significant effect on microbial populations, richness, diversity, and community compositions (Glassman et al., 2017; Kaiser et al., 2016). Ammonia oxidizers are regulated by soil pH, as shown in soils irrigated with untreated wastewater along a century‐scale chronosequence (Aguilar‐Rangel et al., 2024), related to a change in ammonia availability; ammonia is pH dependent (pKa = 9.25, 25°C), ionizing to ammonium under low pH conditions, thus causing soil ammonia deficiency and increased AOA populations (Koper et al., 2010; Martens‐Habbena et al., 2009; Prosser & Nicol, 2012). In addition, changes in ammonia oxidizer communities with changing soil pH are likely (Gubry‐Rangin et al., 2011; Nicol et al., 2008), indicating potential eco‐physiological adaptations and a wide range of ammonia‐oxidizing bacterial and archaeal ecotypes across a gradient of soil pH (Zhalnina et al., 2012). Regarding aerial N loss, soil pH increase, due to HCO_3_^−^ production by microbial activity (B. Liu et al., 2014; J. F. Wang et al., 2007), has been associated with decreasing emissions and N_2_O/(N_2_O + N_2_) ratios (Y. Wang, Guo, et al., 2018), attributed to the sensitivity of translation/assembly of N_2_O reductase to low pH (B. Liu et al., 2014). Higher N_2_O emissions in TWW‐irrigated fields were related to changing soil pH and soil NO_3_^−^ content, as drivers of nitrification and denitrification, rather than changes in soil TN and organic carbon content (Chi et al., 2024). Because TWW‐driven pH shifts are themselves inconsistent in direction across sites (above), this same mechanism plausibly explains why the AOA:AOB ratio response to TWW irrigation is itself inconsistent in direction across the studies reviewed in Section 3.1.2, rather than reflecting an independent, unexplained source of nitrifier variability.

#### 3.2.3 Soil moisture and temperature

Soil moisture and temperature are fundamental parameters shaping soil microbiome and N mineralization (Iqbal et al., 2025) and are regulated in TWW‐irrigated soils by irrigation schedule and soil characteristics (Chauhan et al., 2025; Lõhmus et al., 2006). Soil moisture regulates both dehydration and substrate limitation, shaping nitrifiers (Stark & Firestone, 1995), with variable response due to soil conditions (Adair & Schwartz, 2008; Ebrahimi & Or, 2014; Gleeson et al., 2010; Szukics et al., 2010, 2012; G. Wang & Or, 2013). Moreover, moisture regulates total N_2_O emissions, with peak emissions occurring at different WFPS levels depending on fertilizer status: the highest in unfertilized soil at 100% WFPS but the highest in fertilized soil at 60% WFPS (Xue et al., 2012). This is the same TWW × fertilizer interaction on N_2_O already described in Section 3.1.3 (Xue et al., 2012). TWW irrigation‐driven wet–dry cycles interact with soil properties and induce alternate moisture and oxygenation conditions, shaping denitrifying activity (Congreves et al., 2018; Pinto et al., 2021; Velthuis & Veraart, 2022), given that N_2_O production mainly occurs under anoxic/anaerobic soil conditions. These suggest that dose and frequency of TWW irrigation, along with soil water retention capacity, may interact with several other parameters and reshape aerobic and anoxic nitrogen processes.

Soil temperature is a critical determinant of ammonia oxidation, favored by increased temperature, up to 30°C (Szukics et al., 2010). Temperature is discriminant among ammonia oxidizers, with AOA displaying a preference for higher temperatures compared to AOB, due to thermodynamic differences (X. Lu et al., 2018; Taylor et al., 2017). Moreover, temperature‐related shifts in community structure have been observed both for AOA and AOB (Avrahami & Conrad, 2003), but AOB may exhibit higher stability (Stres et al., 2008; Szukics et al., 2010; Tourna et al., 2008). Temperature is also a significant factor for denitrifiers and their activity, as they prefer warmer conditions (Taylor et al., 2017; Velthuis & Veraart, 2022). These also suggest the temperature as a critical factor in agroecosystems receiving TWW interacting with critical factors discussed in this review; responses in temperature regime may differ in TWW‐irrigated soils compared to freshwater irrigation due to preceding changes in soil microorganisms and activities described previously.

#### 3.2.4 Soil salinity

Unlike pH, the salinity response to TWW is markedly more consistent, showing an increase, regardless of treatment level (Table 9). Continuous TWW irrigation led to an increase in soil EC by 16%–26% relative to a conventionally irrigated control over three growing seasons, without reaching levels considered a salinity risk (Sao et al., 2025). This is consistent with the significant increases in soluble salts reported under long‐term TWW irrigation elsewhere (Chauhan et al., 2025). Tertiary treatment caused an increase in EC across different plant × soil combinations in a mesocosm trial (Zolti et al., 2019). Similarly, irrigation with advanced/Grade 1‐A treated RW increased EC significantly versus potable water even at low levels of salinity (Cui et al., 2025); however, an exception was observed from advanced, high‐rate aerobic‐treated sewage irrigation, where salinity did not increase remarkably despite continued irrigation (J. F. Wang et al., 2007). Irrigation with secondary or tertiary irrigation led to accumulation of soluble salts as compared to rainfed control (Chauhan et al., 2025). Secondary TWW resulted in a significant increase in EC at both 27 years (+2.68 dS/m, reclassified as saline, split between non‐sodic and sodic subgroups) and 16 years irrigation (+4.67 dS/m, saline‐non‐sodic) at the same calcareous site (Feki et al., 2025). Also, in a separate secondary TWW, EC varied significantly by depth within the 15 years of irrigation (from 2.30 to 4.14 mS/cm, 0–20 to 20–40 cm), though this reflects depth stratification rather than a direct duration‐ or control‐based comparison (Werheni Ammeri et al., 2023). Along a raw/untreated, EC increased progressively with duration, peaking at 2.36 dS/m by 50 years (Aguilar‐Rangel et al., 2024). WWTP effluent increased significantly in the soils tested, consistent with the effluent's EC being roughly double that of the tap‐water control (Chroňáková et al., 2024). Similarly, in a four‐soil column study, a dose‐response gradient was observed, continuous TWW > alternating TWW/distilled > distilled control, consistent across all soil types, which was attributed directly to the TWW's own EC (L. Lu et al., 2022). Finally, in secondary TWW irrigation and N‐fertilization gradient, the EC was correlated positively to NO_3_^−^ and negatively to soil pH, rising further at higher N rates under RW irrigation (W. Guo et al., 2018), implied also elsewhere (Tzanakakis et al., 2011).

The above indicates that increased soil salinity should be a common effect in soil receiving TWW. The increase may be the result of a gradual buildup of salts in soil, but the range of increase and temporal variation is a matter of several factors, such as TWW dose and frequency, soil water retention capacity, precipitation, and the presence of vegetation. In any case, the increased salinity may threaten soil structure integrity (under high sodium concentration) and increase soil osmotic potential and toxicity phenomena to microorganisms and plants (Rath et al., 2016; Yan et al., 2015). As microorganisms carry out many biogeochemical processes, any salinity‐related change in their communities will have implications on critical biochemical carbon and nutrients (mainly N) cycles and on the overall functioning of the soil, as previously recorded (Haj‐Amor et al., 2022; G. Zhang et al., 2024). Regarding the response of AOA and AOB populations to salinity stress, contrasting results have been observed, with alternate impairments to both nitrifiers (Bello et al., 2019; H. Wang, Gilbert, et al., 2018), showing potentially a parallel effect in NOB communities (X. Li, Wang, et al., 2021; G. Zhang et al., 2024; Zhu et al., 2022). Such an effect may be dependent on soil salinity level and the nitrifiers’ inherent adapting ability (H. Guo et al., 2020; Huang et al., 2022; H. Wang, Gilbert, et al., 2018; G. Zhang et al., 2024). Change at the level of soil salinity may lead to alteration in N_2_O fluxes: higher under low salinity conditions and the opposite in high salinity soils as has be recorded in a previous study (Q. Wei et al., 2018). The effect of salinity might be masked or mediated by other factors (Chi et al., 2024). Notably, none of the TWW‐specific nitrification or denitrification studies reviewed here are directly attributed to salinity as the primary driver, perhaps over organic loading, pH, ammonia availability, and plant species: salinity's downstream effect on N‐cycling microorganisms is well documented in general soil microbiology (above), yet has been isolated as the operative mechanism; a reported correlation between EC and soil NO_3_^−^ under a TWW/N‐fertilization gradient (W. Guo et al., 2018), itself a bivariate association rather than a confound‐controlled mechanistic test, possibly because salinity typically co‐varies and may be confounded by other factors/parameters.

#### 3.2.5 CECs

Accumulation of CECs under TWW irrigation depends on treatment type, compound persistence, and soil properties, rather than following a uniform pattern (Table 9). For example, advanced/Grade 1‐A treatment, TWW‐irrigated soil accumulated a greater variety and higher concentration of pharmaceuticals than a groundwater‐irrigated soil (Koumaki et al., 2025). Across a large‐scale, 445‐field survey (mixed treatment types), soil accumulation was governed primarily by CEC concentration in the irrigation water and by each compound's soil half‐life, with neither SOM nor irrigation duration (median ∼20 years) exerting a significant effect; among four treatment types compared, only soil aquifer treatment (SAT) significantly reduced soil CEC load, while sand filtration and extended reservoir storage did not outperform standard activated‐sludge treatment (Ben Mordechay et al., 2022). WWTP effluent, treatment not stated, four compounds accumulated progressively in soil over the growing season, with a Cambisol retaining substantially higher concentrations than an Arenosol due to greater sorption affinity (Kodešová et al., 2024). Tertiary TWW (after sand filtration + UV), in a controlled dosing trial, led to sharp compound‐specific soil retention: for example, naproxen and diclofenac were fully degraded with no soil accumulation, and five different compounds accumulated in the soil (Denora et al., 2023). Controlled TWW dosing gradient seems also important regarding accumulation of soil antibiotic concentration (Zheng et al., 2023).

Ammonia oxidizers are sensitive to the presence of antibacterial substances, such as antibiotics and/or EDCs, with possible impairment of nitrifiers and their activity. For example, several compounds, such as azithromycin and clarithromycin, which could exert inhibitory effects, have been found in TWW and soils (Kodešová et al., 2024). Antibiotics can affect microbial diversity and denitrification activity: for example, in grassland soils treated with tetracycline, changes in fungi/bacteria ratios and higher N_2_O production were observed, attributed to the inhibitory effects on *nos*Z‐II‐carrying communities (Semedo et al., 2018). Contradictory findings were obtained by a microcosm study due to the addition of different types of antibiotics (DeVries et al., 2015). As with salinity, the mechanistic plausibility of a CECs–nitrogen cycle link is well established in general soil microbiology (above), yet no TWW irrigation study reviewed here has demonstrated a direct link: studies measuring CECs accumulation and studies measuring N‐process outcomes under TWW irrigation remain largely disconnected, reporting each dimension separately rather than as a tested causal pathway. Antibiotic accumulation in higher plants, affecting yield and posing a possible human health risk, was observed (Geng et al., 2022), implying changes in the plant–soil system.

There is evidence that TWW irrigation consistently increases soil microplastics in comparison to nonirrigated controls; however its magnitude and ranking against other water sources vary. For example, secondary TWW microplastic content increased with irrigation duration, from 13.8 to 861.4 items/kg after 24 years of irrigation (∼62‐fold), with significant stepwise increases at every interval (Hattab et al., 2024). Under treatment level not stated, a 90‐field survey found roughly a twofold increase over control at two depths, microplastics accumulated near the surface, and pollution risk correlating with soil OM, TN, and available P (Aydin et al., 2025). However, when compared directly against other irrigation sources (treatment level not stated), TWW showed significantly lower microplastic abundance than both surface water and groundwater irrigation, though still above nonirrigated control, indicating TWW is not necessarily the largest microplastic contributor among common irrigation sources (Saleh et al., 2025).

Microplastics can reach soil–water–plant systems through TWW irrigation, acting as a critical driver of soil properties, microorganisms, microbial activity, and plant species, reshaping directly or indirectly (through soil conditions regulation) nitrogen and carbon processes (J. Li, Yu, et al., 2022). Moreover, microplastics may act as carriers of several contaminants (Afmataj et al., 2023; Anastopoulos et al., 2021; J. Li et al., 2018; Spyridakis et al., 2022). Microplastics could potentially be degraded (mineralized) by microorganisms (Ali et al., 2021), shaping their communities and enzymes’ activities (Shen et al., 2022; X. Sun et al., 2023; L. Wang et al., 2023). Variable effects on nitrifiers and nitrification activity have been reported for various types of microplastics (Gao et al., 2021; Seeley et al., 2020), while polyamide nylon was demonstrated to stimulate nitrification in soil (Ntaviglaki et al., 2024). Moreover, variable effects of microplastics on denitrifiers’ gene abundances have been reported (Gao et al., 2021; Qian et al., 2018; Rong et al., 2021; Seeley et al., 2020; X. Sun et al., 2023). These may or may not be linked to N_2_O emissions (Gao et al., 2021), depending on their amount and characteristics, their interactions with microorganisms, and soil conditions (X. Chen et al., 2023; L. Li, Song, et al., 2020). Microplastics may affect nitrogen processes in soils through responses and feedback by plant species due to accumulation in plant tissues, which can cause pore blockage, reduced light capture, root system damage, and gene expression hindrance, reshaping soil biological and physicochemical properties (J. Li, Yu, et al., 2022). This same disjunction applies to microplastics: their documented, variable effects on nitrifiers and denitrifiers (above) come exclusively from non‐TWW microplastic‐dosing studies, and no study reviewed in this review has traced a microplastic‐driven shift in nitrogen cycling within a TWW‐irrigated field or mesocosm system specifically.

#### 3.2.6 Heavy metals

TWW contains heavy metals, originating from various sources (Hargreaves et al., 2018). Literature on TWW irrigation consistently shows increases in soil heavy metals, but the specific metals, magnitude, and duration dependence vary by site, soil type, and system. For example, Cu, Zn, Mn, and Fe increased after 27 and 16 years of secondary TWW irrigation in a calcareous soil (Feki et al., 2025), while a separate secondary chrono sequence showed C, Zn, and Ni increased after 20 years, but Cd and Cr remained unaffected under cultivation, spiking instead Cr only at an adjacent noncultivated surplus‐discharge site, showing that, besides treatment, land management as separate factor (Mkhinini et al., 2020). Under advanced/high‐rate aerobic treatment, metals did not increase remarkably (J. F. Wang et al., 2007); a further secondary comparison found opposite duration effects by soil type; a calcisol (17 years, furrow) showed only slight surface Cr enrichment with Zn/Cu stocks decreasing, while a fluvisol (4 years, flood) accumulated Cr, Zn, and Cu at a new subsoil horizon (Belaid et al., 2019). A 40–50 years RW (level not stated) irrigation showed that Cd accumulation tracked water Cd concentration directly (W. Chen et al., 2008). Also, in India, long‐term irrigation with raw/under‐treated wastewater increased Cr, Ni, Cd, and Pb, and Cr and Pb exceeded the limits. A Delhi 5, 10, and 20 years gradient showed metal‐specific responses, with Zn rising progressively, Fe peaking at just 5 years, and then declining, and Ni declining from 10 to 20 years, with Pb plateauing from 10 years, with the same pH drop and organic carbon increase mechanism increasing bioavailable Zn and Cu. Under stabilization‐pond‐treated water, Zn, Cu, Cd, Ni, and Pb increased from 41% to 178% after more than 10 years, with only Cd exceeding the limits (Rezapour et al., 2019).

Accumulated heavy metals interact with the water–soil–microbe–plant system (Sao et al., 2025; Zheng et al., 2023), impacting soil microorganisms, plant species, and animals. Toxic effects of heavy metals on soil microorganisms are due to critical changes in microbial cells, such as binding to active sites, oxidative stress, and molecular inhibition phenomena (Opande et al., 2025). Soil microorganisms are exposed to heavy metal presence, reshaping their populations, communities, and functions involved in nitrogen and carbon cycling (Campillo‐Cora et al., 2025; Tian et al., 2025) depending on edaphic conditions (Opande et al., 2025). This effect has implications for nitrogen cycling processes driven by specialized soil microorganisms. For example, long‐term heavy metal pollution has impaired potential nitrification and denitrification rates, accompanied by changes in nitrifying and denitrifying gene populations, such as *amo*A, *nxr*B (nitrite oxidoreductase β subunit gene), *nir*K (L. Lu et al., 2022). Previously, under treated (non‐raw) long‐term TWW irrigation with domestic wastewater, with BOD_5_/COD values consistent with secondary‐treatment‐range effluent, heavy metal presence was associated with taxon‐specific community shifts rather than uniform suppression, consistent with a gene‐specific rather than uniformly suppressive effect. In addition, variation in N‐transforming gene abundances (*amo*A, *nif*H [nitrogenase iron protein gene], *nir*K, and *nir*S) was explained by heavy metal accumulation rather than soil physicochemical factors or depth (28.4% vs. 13.3% and 8.4%), suggesting that heavy metals may reshape the balance between N‐transforming gene pools rather than depress them uniformly (Xi et al., 2021). Also, in a 5‐ to 25‐year irrigation with secondary TWW, a decline in total bacterial density alongside a taxonomic shift was observed (decrease in Actinobacteriota, Bacteroidota, and Firmicutes and increase in Acidobacteriota, Patescibacteria, and Gammaproteobacteria), attributed by the source to the combined pressure of TWW‐associated stressors (i.e., salinity, acidification, carbon input, and antibiotics) rather than exclusively to heavy metal accumulation; only the parallel, duration‐dependent increase in metal‐resistant bacterial populations was specifically and directly attributed to heavy metal accumulation, indicating that heavy metal pressure reshapes resistance profiles, even where its contribution to broader community shifts, or to nitrogen‐transforming gene abundance, was not isolated (Yagoubi et al., 2024). Regarding plant species, under secondary/simulated antibiotic dosing, metal distribution shifted with co‐contamination, being lower in soil/roots but higher in shoots/grain at the highest dose, via antibiotic‐metal complexation (Zheng et al., 2023). The last two references indicate the interaction between the heavy metal and specific CECs pressures, potentially leading to changes in microorganisms, plant species, and nitrogen processes.

#### 3.2.7 Soil type, land use, and field practices

Soil type, through its effect on active area and retention capacity, medium porosity, water‐holding capacity, and aeration, is a critical parameter for N processes under TWW irrigation (Table 10). Higher nitrifier abundances and activities have been observed in clayey soils than in sandy soils; however, there is no clear discrimination between AOA and AOBs (de Gannes et al., 2014; Fortuna et al., 2012; Pereira e Silva et al., 2012). Different land use types and agronomic practices reasonably regulate the shaping of nitrifiers (Keil et al., 2011; Le Roux et al., 2007; A. Meyer et al., 2014; Taylor et al., 2010; Wessen et al., 2011; Zeglin et al., 2011). Observed effects, however, were not consistent, indicating co‐effects of several critical parameters (Bru et al., 2011; Yao et al., 2013). Direct TWW‐specific evidence confirms soil type as an independent, sometimes dominant driver: under identical irrigation, ammonium and nitrate accumulation was governed by soil type (Cambisol > Arenosol), with the CEC and NO_3_^−^ response concentrated almost entirely in one soil (Chroňáková et al., 2024; Kodešová et al., 2024). Soil type can even reverse duration effects, and a calcisol under 17‐year irrigation showed declining metal stocks, while a fluvisol under only 4 years accumulated metals at a new subsoil horizon (Belaid et al., 2019), indicating soil type can be as decisive as water quality itself.

**TABLE 10:** Changes of specific parameters in soils under treated wastewater (TWW) irrigation, acting as potential drivers of nitrogen processes.

Category	Driver	Treatment level	Finding	Direction	Source
OM/DOC/TN	40 years, secondary→tertiary	Secondary→tertiary	OM and TN accumulated significantly	↑	Chauhan et al., 2025
	Mesocosm	Secondary	Pore‐water DOC significantly ↑	↑	Zolti et al., 2019
	27 years and 16 years calcareous plots	Secondary	OM ↑ both durations, no diff between them	↑	Feki et al., 2025
	Chronosequence 0/1/7/15 years	Secondary	TOC/TN/OM ↑ progressively with duration; onset varies by depth and parameter—N increased already at 1 year across all depths, while OM/C showed delayed onset (no change at 1 year) only in the deepest layer (40–60 cm)	↑	Werheni Ammeri et al., 2023
	Citrus orchard/arable, 2 sites	Secondary	SOC lower under TWW (*p* = 0.07; *p* = 0.05)	↓	Heinze et al., 2014
	Four‐level lysimeter	FW/secondary/tertiary/UF	Texture‐driven, not water‐quality; only P ↑	Mixed	Heinze et al., 2014
	>20 years, Mallorca	Secondary	Bulk OM no diff; DOC significantly ↑	↑ (DOC only)	Adrover et al., 2012
	4 years persimmon orchard	Secondary (upgraded 2007)	DOC ↑ vs. N‐matched FW, diminished post‐upgrade	↑ → diminishing	Elifantz et al., 2011
	153‐day soil column	Tertiary	No significant TOC diff vs. matched synthetic FW	No effect	Ibekwe et al., 2018
	Single season, 2 soils	Not stated	Cox and DOC ↓ (priming effect); NO_3_^−^ ↑	↓ (Cox/DOC), ↑ (NO_3_^−^)	Chroňáková et al., 2024
pH	40 years comparison	Secondary→tertiary	7.43–7.98 vs. 5.05–6.04	↑	Chauhan et al., 2025
	Century‐scale chronosequence	Raw/untreated	7.70→8.4–8.5	↑	Aguilar‐Rangel et al., 2024
	Mesocosm, 4 combos	Secondary	+0.1 to +0.9, *p* < 0.0001	↑	Zolti et al., 2019
	27 years/16 years plots	Secondary	+0.33/−0.54 units	↑/↓	Feki et al., 2025
	>20 years, Mallorca	Secondary	+0.3, *p* < 0.001	↑	Adrover et al., 2012
	18‐month TSE, Brazil	Secondary	4.8→5.5	↑	de Paula et al., 2009
	Mediterranean LTS	Primary	Delayed, topsoil‐only	↑ (delayed)	Tzanakakis et al., 2011
	Chronosequence 0/1/7/15 years	Secondary	8.30→7.81 by 15 years	↓	Werheni Ammeri et al., 2023
	Loess soil, China	Secondary	8.39→8.05, judged insignificant	↓ (small)	J. F. Wang et al., 2007
	Sludge + RW pot trial	Advanced/Grade 1‐A	7.98→7.66, further ↓ with sludge	↓	Cui et al., 2025
	N‐fertilization gradient	Secondary	RW < CW at matched N	↓	W. Guo et al., 2018
	Maize/lavender/Oved/Truu	Advanced‐tertiary/Secondary	No significant difference (×3)	No effect	Mola et al., 2024; Oved et al., 2001; Truu et al., 2009
Salinity/EC	Mesocosm	Secondary	Significantly ↑, doubled in 3/4 combos	↑	Zolti et al., 2019
	40 years comparison	Secondary→tertiary	159.66 vs. 19.86 µS/cm	↑	Chauhan et al., 2025
	27 years and 16 years plots	Secondary	+2.68 and +4.67 dS/m	↑	Feki et al., 2025
	Sludge + RW pot trial	Advanced/Grade 1‐A	243→358 µS/cm, further ↑ with sludge	↑	Cui et al., 2025
	4‐soil column, dose gradient	Not stated	Continuous > alternating > distilled	↑ (dose‐response)	L. Lu et al., 2022
	Century‐scale chronosequence	Raw/untreated	Peaked 2.36 dS/m by 50 years	↑	Aguilar‐Rangel et al., 2024
	N‐fertilization gradient, tomato	Secondary	EC + with NO_3_^−^, − with pH; ↑ further at higher N under RW	↑	W. Guo et al., 2018
	Loess soil, China	Secondary	Did not increase remarkably	No effect	J. F. Wang et al., 2007
CECs/ARGs	GW vs. TWW, maize field	Advanced/Grade 1‐A	Greater CEC variety/concentration	↑	Koumaki et al., 2025
	445 fields, Israel	Mixed (only SAT ↓)	Water‐driven, not OM/duration‐driven	Mixed	Ben Mordechay et al., 2022
	Raised beds	Not stated	4 compounds accumulated progressively	↑	Kodešová et al., 2024
	Lysimetric dosing, tomato	Tertiary	Compound‐specific, up to 100% soil‐retained	Compound‐specific	Denora et al., 2023
	Microcosm, antibiotic gradient	Secondary (simulated)	ARGs ↑ up to 328%, *sul1* dominant	↑	Zheng et al., 2023
	*ermB*/*sul1*, rhizosphere	Advanced/Grade 1‐A	Significantly ↑	↑	Cui et al., 2025
Microplastics	Duration gradient (5/16/24 years)	Secondary (activated sludge, medium load, since 1993)	Control: 13.8 → 5 years: 338.6 → 16 years: 555.6 → 24 years: 861.4 (∼62‐fold over 24 years)	↑ (monotonic)	Hattab et al., 2024
	90 fields vs. control	Not stated	Control: 169 ± 46.8 (0–10 cm), 140 ± 44.7 (10–20 cm) → TWW: 329 ± 139.5 (0–10 cm), 295 ± 115.4 (10–20 cm)—roughly twofold increase, *p* < 0.0001.	↑	Aydin et al., 2025
	TWW vs. other sources	Not stated	TWW significantly Lower than surface/groundwater	↑ vs. control, ↓ vs. others	Salehi et al., 2023
Heavy metals	27 years and 16 years plots	Secondary	Cu/Zn/Mn/Fe ↑	↑	Feki et al., 2025
	20 years chronosequence	Secondary	Cu/Zn/Ni ↑; Cd/Cr unaffected (cultivated)	↑ (selective)	Mkhinini et al., 2020
	Loess soil, China	Secondary	Did not increase remarkably	No effect	J. F. Wang et al., 2007
	Calcisol (17 years) vs. fluvisol (4 years)	Secondary	Opposite by soil type/duration	↓/↑	Belaid et al., 2019
	Cd, 40–50 years, 2 RW sources	Not stated	Tracks water Cd concentration	↑ (water‐dependent)	Chen et al., 2013
	Central India, >20 years	Likely raw/under‐treated	Cr/Ni/Cd/Pb ↑, Cr and Pb exceed limits	↑	Prasad et al., 2026
	Delhi, 5/10/20 years gradient	Likely raw/under‐treated	Metal‐specific, non‐monotonic	Mixed	Rattan et al., 2005
	Stabilization ponds, >10 years	Secondary‐equivalent	Zn/Cu/Cd/Ni/Pb ↑ 41%–178%; Cd exceeds limit (source inconsistent on whether Cd or Cu shows the 178.2% peak)	↑	Rezapour et al., 2019
	Antibiotic‐dosing rice	Secondary (simulated)	↓ soil/roots, ↑ shoots/grain at high dose	Redistributed	Zheng et al., 2023
	DTWW/ITWW, 30 years, N‐transforming genes	Treated, not explicitly labeled (BOD/COD consistent with secondary‐range)	HMs explain 28.4% N‐gene variance and 21.5% community variance (>physicochemical factors/depth); ITWW ↑*amo*A all depths, ↓*nifH*; taxon‐specific shifts (Micrococcaceae↔HM+, Bacillus↔HM−)	Gene‐specific redistribution, not uniform suppression	Xi et al., 2021
	5 years and 25 years gradient, resistome/community	Secondary (activated sludge, confirmed)	↓ total density; ↑ metal/antibiotic‐resistant populations (duration‐dependent); taxonomic shift attributed to combined stressors, not HM alone; N‐cycling genes not assessed	↓ density, ↑ resistance (selective)	Yagoubi et al., 2024

Abbreviations: ARGs, antibiotic resistance genes; BOD, biochemical oxygen demand; CECs, contaminants of emerging concern; COD, chemical oxygen demand; CW, control water (deionized water); DOC, dissolved organic carbon; EC, electrical conductivity; FW, freshwater; GW, groundwater; HM, heavy metal; LTS, land treatment system; OM, organic matter; RW, reclaimed water; SAT, soil aquifer treatment; SOC, soil organic carbon; TN, total nitrogen; TOC, total organic carbon; TSE, treated sewage effluent; UF, ultrafiltration.

#### 3.2.8 Plant species

Soil and vegetation response to TWW irrigation is shaped substantially by plant species itself, particularly the tree/perennial‐versus‐annual distinction, sometimes with an effect size exceeding that of water quality itself. Across a 445‐field survey (mixed treatment levels, including SAT), the leaf accumulation factor of three frequently detected pharmaceuticals was consistently and significantly lower in trees (citrus and avocado) than in annual crops (tomato, potato, carrot, and leafy greens) grown in the same soil and irrigated with the same water (e.g., carbamazepine leaf accumulation factor [LAF] of 0.55 in citrus vs. 92.5 in tomato, attributed to differing plant physiology and in‐planta processing between trees and annuals) (Ben Mordechay et al., 2022). Crop type also shaped soil enzymatic profile independently of water quality: β‐glucosidase dominated in a citrus orchard while amino‐peptidases dominated at two arable sites, regardless of irrigation water source at each site (Heinze et al., 2014). Nitrification response likewise varied by plant type under the same water: under primary treatment, the potential nitrification rate rose 155% under a tree species (*Eucalyptus camaldulensis*) versus 250% under a nonwoody reed (*Arundo donax*) (Tsiknia et al., 2013). Perennial vegetation has also acted as a confounding factor in biomass findings: under secondary treatment, microbial biomass increased equally in irrigated and nonirrigated willow‐coppice plots, with the increase attributed by the source itself to willow establishment rather than water quality (Truu et al., 2009), and perennial alfalfa cropping similarly acted as a potential contributor to elevated DOC after more than 20‐year secondary TWW irrigation, independent of TWW itself (Adrover et al., 2012). Finally, heavy‐metal accumulation capacity differed markedly across 14 crop species grown in the same likely raw/under‐treated irrigated soil, from suppressed uptake in sorghum to elevated uptake across nearly all measured metals in maize (Rattan et al., 2005). These indicate the polyvalent role of plant species in shaping various drivers’ effects and their interactions in TWW‐irrigated soils, overcoming in some cases the TWW quality‐driven effects.

#### 3.2.9 Persistence and reversibility of driver effects

The persistence and magnitude of the effects discussed above depend not only on TWW but also on irrigation duration and seasonality. Irrigation duration was explicitly tested, highlighting that microbial responses tend to intensify with the time of irrigation, with responses being indicator‐specific. For example, a chrono sequence spanning 1, 7, and 15 years of secondary‐treated wastewater irrigation caused an increase in heterotrophic bacteria and filamentous fungi, an effect that emerged only after the seventh year, while microbial biomass C and N emerged after the first year (Werheni Ammeri et al., 2023). Similarly, other studies, such as 0‐ to 15‐year study, showed consistent duration‐dependent stimulation of heterotrophic bacteria, fungi, and microbial biomass C (along with soil respiration) (Hidri et al., 2018), or 100‐year TWW‐irrigated soils showing, within organic soil particle fractions, an increase in bacteria, actinomycetes, and fungi compared to nonirrigated soil (Filip et al., 2000). Finally, 20 years or more of TWW irrigation was found to have higher microbial biomass relative to non‐TWW‐irrigated soil, although the comparison was non‐paired and non‐contemporaneous and therefore weaker evidence than the chrono sequence designs described above (Adrover et al., 2012). However, it seems that the duration factor is not unconditional or automatically cumulative and may display seasonal variations dependent on the irrigation period. Irrigation duration is also an important source of variation and can be transient, from increasing to baseline or lower values (Elifantz et al., 2011; Y. Zhou et al., 2025). This indicates that, at least on this timescale and in this system, seasonal irrigation produced a reversible, within‐season response rather than a persistent, accumulating shift in soil microbial activity, a distinction that should be made explicit whenever driver effects from different studies are compared. However, long‐term inputs can have a persistent legacy: enzyme activities and microbial counts yet remained detectable even 20 years after irrigation had ceased (Filip et al., 2000). Taking together the available evidence, it is suggested that treatment potential and time are crucial to soil microorganisms and their activity, a distinction the existing literature synthesis has generally not made explicit.

Overall, the drivers examined in this section do not act uniformly, independently, or with equal evidentiary support. TWW‐irrigated soils experience simultaneous shifts in organic loading, pH, salinity, contaminant burden, and metal content, yet only a subset of these, such as organic loading and pH, have been mechanistically linked to specific nitrogen‐process outcomes within TWW‐specific studies; for salinity, CECs, and microplastics, comparable links remain either weakly supported or entirely unexplored in the field. On this driver‐level variability, plant species and soil type repeatedly emerge as confounding or dominant factors capable of exceeding water‐quality effects altogether, and the persistence of any given driver's effect is indicator‐specific rather than uniformly cumulative; some responses are seasonally reversible, others are detectable decades after irrigation ceases. This combination of multi‐driver co‐variation, uneven mechanistic evidence, and confounding by plant and soil factors is the central methodological challenge for interpreting and comparing the nitrogen‐cycle findings reviewed in Section 3.1.

## 4 WHAT WE KNOW: SYNTHESIZING CURRENT KNOWLEDGE, MAJOR KNOWLEDGE GAPS, AND RESEARCH NEEDS

### 4.1 What we know: A synthesis of the current knowledge (summary)—Knowledge gaps and research needs

Across nearly every parameter reviewed, the literature does not support a single, uniform WW effect. Outcomes are consistently conditional on treatment level, irrigation duration, soil type, plant species, and co‐applied fertilization, and studies that control for one or more of these factors repeatedly show that an apparent TWW effect can shrink, disappear, or reverse once the relevant confound is accounted for. This context‐dependency is itself the principal finding of the reviewed evidence base. The strength of mechanistic evidence linking specific drivers to specific nitrogen‐process outcomes is itself uneven. The main knowledge gained here is summarized in Table 11. In addition, the major knowledge gaps in TWW irrigation are categorized and summarized as follows in Table 12.

**TABLE 11:** Synthesis–summarization of the current knowledge on treated wastewater (TWW) irrigation and N cycling.

Synthesis—Summarization	Key references
Microbial biomass, diversity, and community composition respond to TWW irrigation, but neither direction nor magnitude is uniform; treatment level is a major but not sufficient determinant, and site‐specific factors (soil type, land‐use history, co‐irrigated crop) can override an apparent TWW effect.	Heinze et al., 2014; Lal et al., 2015; Moulia et al., 2023; Rosin et al., 2020; Truu et al., 2009
Bacterial and fungal communities frequently diverge in their response to TWW within the same study: one taxonomic group changes while the other does not, or the two diverge by depth or over time.	Chauhan et al., 2025; Dang et al., 2019
Soil enzyme activity is, on balance, stimulated relative to freshwater or nonirrigated controls, but the response is enzyme‐specific and depends on treatment level and duration of irrigation.	W. Chen, Wu et al., 2008; Heinze et al., 2014; Y. Zhou et al., 2025
Nitrification is neither consistently stimulated nor consistently inhibited. AOA and AOB frequently respond differently from one another, and the direction of that divergence is itself inconsistent across studies; a shift in nitrifier community composition does not reliably predict a shift in nitrification activity. Inhibition, via organic‐load‐driven O_2_ competition and/or CECs, has also been documented. Stronger stimulation under raw/untreated wastewater rests on a single available study, not yet a replicated pattern.	Aguilar‐Rangel et al., 2024; Chi et al., 2023, 2025; Chroňáková et al., 2024; Cui et al., 2025; Master et al., 2003
Functional redundancy can preserve nitrification activity even where nitrifier community composition and genetic diversity change.	Lopes et al., 2016
Comammox bacteria have received TWW‐specific attention in at least two studies (family‐level abundance shifts; soil/time‐specific declines), but remain markedly less characterized than AOA/AOB, not “overlooked” outright.	Chauhan et al., 2025; Chroňáková et al., 2024
NOB (nitrite‐oxidizing bacteria) responses are comparably under‐characterized: where reported alongside AOA/AOB, NOB abundance is consistently found unaffected but is never the focus of dedicated analysis.	Chi et al., 2023, 2025
Denitrification and N_2_O emissions generally increase under TWW irrigation, together with denitrification‐gene abundance (*nir*K, *nir*S, *nos*Z), but the magnitude depends strongly on co‐applied fertilization, fertilizer type, and soil moisture status. Nitrification itself is an important, sometimes dominant, contributor to N_2_O production, not merely a precursor to denitrification.	Chi et al., 2023; Master et al., 2003; Xue et al., 2012, 2024
NH_3_ volatilization is jointly modulated by soil pH and co‐applied fertilization, with the same TWW input capable of decreasing or increasing losses depending on fertilizer co‐application.	M.‐Y. Li, Wang, et al., 2020; Master et al., 2003
Inorganic N forms (NO_3_^−^, NH_4_^+^, NO_2_^−^) typically do not move together; they frequently respond independently, sometimes in opposite directions, depending on water‐source quality, soil type, depth, and plant species.	Chi et al., 2024; Chroňáková et al., 2024; Junejo et al., in press; Livesley et al., 2007
Organic carbon and nitrogen loading is a plausible unifying mechanism behind several of the above patterns, but its own effect on SOM and DOC is not uniform; increases, no change, and decreases are all reported under comparable treatment levels, with duration and soil type determining which pattern is observed, plausibly explaining, at least in part, why organic‐load‐driven nitrification inhibition is reported in only some of the TWW studies reviewed here, rather than reflecting an independent source of unpredictability.	Chauhan et al., 2025; Elifantz et al., 2011; Feki et al., 2025; Heinze et al., 2014
Soil pH change is inconsistent in direction and does not track treatment level in any simple way. Where a mechanism is identified, it is consistently geochemical (cation exchange, carbonate/CO_2_ equilibrium) rather than nitrogen‐cycle‐mediated, and pH in turn regulates ammonia availability and nitrifier community composition, plausibly explaining, at least in part, why the AOA:AOB divergence reported elsewhere in this synthesis is itself inconsistent in direction across studies, rather than reflecting an independent source of unpredictability.	Aguilar‐Rangel et al., 2024; Feki et al., 2025; W. Guo et al., 2018; Oved et al., 2001; Sao et al., 2025
Soil moisture and temperature regulate nitrification and denitrification through established general mechanisms, but this driver is evidenced mainly through general soil‐microbiology principles rather than TWW‐specific dose–response studies.	Szukics et al., 2010; Taylor et al., 2017
Soil salinity (EC) is the most consistently affected property, increasing across nearly all treatment levels and systems, with only isolated exceptions. Despite this consistency at the EC level, individual ion accumulation varies markedly and sometimes counterintuitively by soil type.	Chauhan et al., 2025; Sao et al., 2025; Lu et al., 2022; J. F. Wang et al., 2007 (exception)
Pharmaceuticals and antibiotics accumulate in TWW‐irrigated soil in a compound‐specific, persistence‐driven manner; the largest available survey found no significant effect of soil organic matter or irrigation duration, with soil concentration not reliably tracking irrigation‐water concentration.	Ben Mordechay et al., 2022; Denora et al., 2023; Koumaki et al., 2025
Antibiotic resistance genes (ARGs) increase in TWW‐irrigated soil under both simulated dosing (*sul1*, up to 328%) and real‐field irrigation (*ermB*/*sul1* in rhizosphere soil); heavy metal co‐contamination can independently reshape N‐transforming and resistance gene pools rather than uniformly suppressing them.	Zheng et al., 2023: Cui et al., 2025; Xi et al., 2021
Soil microplastics increase consistently with TWW irrigation duration relative to nonirrigated controls, but TWW is not necessarily the largest microplastic contributor relative to other irrigation water sources.	Aydin et al., 2025; Hattab et al., 2024; Saleh et al., 2025; Salehi et al., 2023
Heavy metals accumulate under TWW irrigation in most systems examined, but which metals, at what magnitude, and over what timescale is highly site‐ and metal‐specific; land management can act as an independent factor alongside treatment level and duration.	Feki et al., 2025; Mkhinini et al., 2020; Rezapour et al., 2019; Xi et al., 2021
Soil type and land use condition the TWW response at every level discussed above, but the literature isolating soil type as an independent driver (rather than a confound within another comparison) remains comparatively thin.	Bru et al., 2011; de Gannes et al., 2014; Fortuna et al., 2012
Plant species is a major, sometimes dominant, determinant of TWW effects on soil nitrogen processes, in several cases with an effect size exceeding that of water quality itself; perennial vegetation has repeatedly acted as a confounding driver behind biomass or DOC findings otherwise attributed to TWW.	Ben Mordechay et al., 2022; Truu et al., 2009; Tsiknia et al., 2013; Adrover et al., 2012
Duration and persistence effects are indicator‐specific rather than uniformly cumulative: some responses intensify progressively and leave a detectable legacy after irrigation ceases, while others are seasonally reversible.	Elifantz et al., 2011; Filip et al., 2000; Werheni Ammeri et al., 2023

Abbreviations: AOA, ammonia‐oxidizing archaea; AOB, ammonia‐oxidizing bacteria; CECs, contaminants of emerging concern; DOC, dissolved organic carbon; EC, electrical conductivity; SOM, soil organic matter.

**TABLE 12:** The major knowledge and methodological gaps in treated wastewater (TWW) irrigation.

**Wastewater treatment—Removal of CECs**	
Secondary vs. tertiary reclaimed waters differ in DOC, NH_4_^+^ and micropollutant loads, so outcomes for soil N cycling vary widely with treatment train and wastewater source.	(Section 2.1)
There is a need to develop integrated decision‐support tools accounting for TWW treatment level regarding nitrogen and CECs, soil properties and conditions, and irrigation and fertilization adjustments.	(Section 2.1)
**Wastewater treatment—Removal of CECs** **N mineralization processes—Enzyme activity, priming effect, and CECs**	
Mechanistic studies linking specific soil‐enzyme kinetics (e.g., urease, protease) to actual nitrogen mineralization rates under the elevated and compositionally distinct organic loading typical of TWW remain scarce, limiting the translation of enzyme‐activity findings into process‐rate prediction.	(Section 3.1.1)
Relative contribution and rates partitioning between bacterial and fungal decomposers to depolymerization under TWW irrigation are rarely disentangled.	(Chauhan et al., 2025)
The potential “priming effect” of soil (mineralization of native organic matter or N), which can be accelerated or suppressed by TWW—added labile organic C and inorganic N, remains unknown.	(Jueschke et al., 2008)
Effect of CECs on soil microbiome and functioning, including enzyme activity and soil N process rates.	(Aydin et al., 2025; Capodaglio, 2017; Yakamercan et al., 2025)
No study reviewed here traced a CEC or ARG signal across the full soil‐water‐crop continuum within a single experimental design—each stage of the exposure pathway is currently evidenced by a different, nonoverlapping body of literature.	–
Integrated environmental risk assessment (ERA) frameworks, evaluating CECs, ARB/ARGs, soil health, groundwater quality, crop contamination pathways, and ecosystem impacts jointly rather than as separate endpoints, are largely absent from TWW‐specific research	(European Commission [EC‐COM], 2018; Pazda et al., 2019)
**Nitrification processes—Comammox, NOB, irrigation and temperature regime, and CECs**	
Comammox's role in nitrification and denitrification, and comammox‐specific N_2_O yield, are largely unknown (Chauhan et al., 2025; Séneca et al., 2020).	(Chauhan et al., 2025; Séneca et al., 2020
Nitrite oxidizers (NOB) (e.g., Nitrospira, Nitrobacter) responses and nitrite kinetics under TWW irrigation are commonly omitted from nitrification studies.	(Chi et al., 2023, 2025)
Linking nitrifier gene/transcript abundance to actual gross nitrification rates is a general methodological gap, but it is particularly acute in TWW‐irrigated soils, where organic loading, salinity, and CECs can decouple gene abundance from activity (Section 3.1.2) more than in conventionally irrigated soils, yet very few TWW studies pair molecular and flux measurements.	(Johnston et al., 2023; Séneca et al., 2020; Wankel et al., 2011)
CECs' potential to inhibit nitrogen processes (e.g., responses of AOA and AOB) and recovery potential.	(Yakamercan et al., 2025)
TWW irrigation can create transient anaerobic/anoxic or low O_2_ conditions in soil due to dry–wet cycles. The resilience and kinetics of nitrifiers' activity, including comammox, in this fluctuating redox regime are poorly known.	(Ribeiro et al., 2024; Séneca et al., 2020)
Temperature and soil pH, despite the available knowledge, need to be evaluated in TWW‐irrigated soils to explore how TWW‐induced shifts in pH and thermal regime modify nitrifiers and their activities.	(Séneca et al., 2020; Taylor et al., 2017)
TWW‐driven soil salinity and its pulses can affect nitrification processes and partitioning among nitrifiers. More studies in different soil types and conditions are needed.	(Aguilar‐Rangel et al., 2024; Bello et al., 2019)
Given that TWW irrigation is documented in this review to increase N_2_O emissions (Section 3.1.3), soil salinity (Section 3.2.4), and CEC/heavy‐metal accumulation (Section 3.2.5–3.2.6), whether soil amendments such as biochar, nitrification inhibitors, or biostimulants can specifically offset these TWW‐associated impacts, rather than serving a generic soil‐management function, has not been examined.	(X. Liu et al., 2021; Ribeiro et al., 2024).
**N_2_O generating processes: Partitioning, comammox, irrigation and redox regimes, transcripts, and salinity**	
N_2_O partitioning among N_2_O‐producing processes is rarely investigated in TWW research	(Xue et al., 2012)
The role of comammox in N_2_O fluxes in TWW‐irrigated soil remains unexplored. Under transient regimes of pH, moisture, or inhibition, comammox N_2_O yields might diverge.	(Pan et al., 2023; Séneca et al., 2020)
Study of the temporal pulses due to TWW irrigation events and consequences in N_2_O‐producing processes. Studies on high‐frequency monitoring under different soil properties are needed.	(Ribeiro et al., 2024)
Except for quantification of functional genes (*nir*k, *nir*S, and *nos*Z abundances), we need to examine transcriptional (mRNA) dynamics to enhance the mechanistic link with denitrifying communities and observed N_2_O fluxes in TWW‐irrigated soils.	(Drewer et al., 2020; S. Meyer et al., 2021)
Salinity and ionic strength should directly or indirectly affect denitrification kinetics and the N_2_O/(N_2_O + N_2_) ratio. TWW reasonably raises salinity and ionic strength, which can inhibit denitrifiers; little is known so far.	(Pan et al., 2023).
Studies are needed to elucidate the interaction between TWW labile C, irrigation regime‐aeration gradients, denitrifying communities, and N_2_O fluxes.	
TWW irrigation and CEC inputs to soil can inhibit N_2_O processes. We need studies to elucidate this effect, focusing on critical factors and the potential distinct responses among denitrifiers.	(Yakamercan et al., 2025)
**Ammonia volatilization**	
Ammonia volatilization driven by externally induced pulses in soil pH is an underestimated nitrogen loss that needs further investigation in TWW‐irrigated soils. High‐resolution field measurements of NH_3_ fluxes following TWW irrigation are rare.	(W. Yang et al., 2020)
How TWW‐driven shifts in NH_4_^+^ availability and soil pH jointly regulate ammonia volatilization, microbial/plant NH_4_^+^ uptake, and nitrification remains unaddressed as an integrated system; most studies measure these processes separately rather than as competing sinks for the same TWW‐supplied NH_4_^+^ pool.	(Section 3.2.2) (Section 3.1.3)
TWW directly (content) or indirectly (microbial activity) may create microgradients in soil pH and affect volatilization kinetics.	(Merl et al., 2024)
**Plant species responses—NUE** **and WUE, plant‐soil microbiome interaction, N loss, CECs, salinity**	
Whether the species‐specific differences already documented under TWW irrigation, divergent nitrification response by species (Section 3.2.8), and divergent contaminant leaf accumulation between trees and annuals (Section 3.2.5) translate into meaningful differences in N‐use efficiency (NUE) or water‐use efficiency (WUE) has not been tested.	Ben Mordechay et al., 2022; Tsiknia et al., 2013
The effect of salinity, ionic strength, and CEC accumulation on plant species and plant‐soil interaction under TWW irrigation is rarely examined.	(Denora et al., 2023)
Given that plant species is repeatedly identified as a dominant, sometimes confound‐inducing, driver of TWW effects, whether species identity similarly modulates the N_2_O and NH_3_ losses documented under TWW irrigation still remains unexplored.	(Section 3.1.3)
Studies are needed on plant species–soil microbiome specificity in TWW‐irrigated cultivations to link plant performance and characteristics with rhizosphere microbiota involved in critical soil N processes and loss.	(Zolti et al., 2019)
New effective models regarding soil microorganisms‐plant species‐water system response and nitrogen recovery and consequences in N budget and assessment of the climate impacts for TWW reuse	(Kefi et al., 2023)
**Methodological gaps in TWW irrigation**	
Landscape‐scale and GIS‐based background characterization. Nitrogen cycling in TWW‐irrigated soils is evaluated almost exclusively by on‐site physicochemical drivers (Section 3.2); however, the surrounding landscape context, catchment‐scale nutrient sources, historical land management, and spatial heterogeneity in background soil properties are rarely characterized before irrigation begins. This is a genuine gap for the same reason that treatment‐level, duration, and soil‐type confounds repeatedly reshape or reverse apparent TWW effects throughout this review (Section 4.1): without a spatially explicit baseline, an observed nitrogen or microbial response may partly reflect pre‐existing landscape controls rather than TWW irrigation itself. GIS‐based and spatially explicit digital soil mapping approaches, increasingly used to characterize regional soil chemical variability and its land‐use drivers, offer a practical route to establishing this baseline and could usefully be incorporated into long‐term TWW field study design.	(Biedunkova et al., 2026)
Long‐term field studies in semi‐arid or arid areas investigating different TWW regimes (quality, frequency and dose), climate characteristics and soil types.	(Masrahi, 2023)
Most studies focus on 0–20 cm of the soil profile; deeper sampling will help in better understanding of the legacy effect and nature of soil.	(Masrahi, 2023)
Legacy effects and cumulative impacts on nitrogen cycling after a long period of TWW application are needed, focusing on micropollutants, alteration of soil microbiome, and changes in soil organic C and its fractions.	(Shi et al., 2025)
High‐frequency and seasonal sampling covering pulses (e.g., dry–wet cycles), transient responses, and seasonal variations are necessary, especially for GHGs and microbial changes and activities, particularly in semi‐arid regions, such as the Mediterranean climatic regime.	(Ribeiro et al., 2024)
Studies on microbial resilience and functional redundancy will describe the recovery potential of soil microorganisms in TWW‐irrigated lands.	(Shade, 2023)
Current advances in molecular biology techniques have made progress in early detection and identification of antibiotics and ARGs; however, work should be done on quantitative analysis of environmental samples and on the development of unified methodologies to increase CECs traceability in the soil‐water‐plant matrix.	(Karkman et al., 2018);
Since organic C and N loading under TWW is itself highly inconsistent across studies rather than a fixed, predictable input, irrigation‐scheduling models calibrated for conventional water‐only inputs may not transfer reliably to TWW systems without explicitly parameterizing this loading variability.	(Section 3.2.1)
Given the number of interacting, confounding factors identified throughout this review as jointly determining TWW outcomes (treatment level, duration, soil type, plant species—Section 4.1), machine‐learning approaches capable of handling high‐dimensional, nonlinear interactions may offer a more tractable path to predictive modeling than single‐factor experimental designs alone.	(Köksal et al., 2025)
Environmental sustainability trade‐offs: TWW irrigation reduces freshwater abstraction and wastewater discharge, and its nutrient content can offset synthetic fertilizer use, but no study reviewed here places these conservation benefits in a common accounting framework against the soil‐ and atmosphere‐level costs identified in this review (N_2_O emissions, salinization, and CEC accumulation sections).	–
Life‐cycle and integrated risk‐sustainability assessment approaches, evaluating soil, groundwater, crop, and ecosystem endpoints together, alongside water and carbon conservation gains, remain largely unexplored for TWW irrigation in agricultural systems.	(European Commission [EC‐COM], 2018).

Abbreviations: AOA, ammonia‐oxidizing archaea; AOB, ammonia‐oxidizing bacteria; ARB, antibiotic‐resistant bacteria; CEC, contaminants of emerging concern; DOC, dissolved organic carbon; GHGs, greenhouse gases; GIS, geographic information system; mRNA, messenger RNA; NOB, nitrite‐oxidizing bacteria.

### 4.2 Scientific questions and conceptual gaps in TWW irrigation

#### 4.2.1 Microbial mediation and changes in functional redundancy and plasticity in TWW irrigation lands?

Short‐or long‐term TWW irrigation may mediate soil microbial communities, although such an effect varies, as shown in this review. The legacy and time effects, the TWW regime, soil types, plants, and native microbial communities are crucial in shaping the soil microbiome and its function. However, it remains unknown whether potential changes in soil microbial community due to TWW irrigation have consequences on microbial functional redundancy and plasticity, both associated with ecosystem performance and resilience to various stressors derived from TWW; Ιt is known that soil microorganisms, due to genomic plasticity, can adjust to new conditions, being also faster‐adapting than higher eukaryotes (Bang et al., 2018). This microbial adjustment to a new equilibrium can either allow soil to continue to function (Schloter et al., 2018) or furthermore contribute to plant survival under challenging conditions such as TWW irrigation (Astudillo‐García et al., 2019). A reasonable question in that case is the duration and persistence of this change, as well as what are the underlying TWW‐related parameters/factors (e.g., CECs) (Gomes, 2025), discussed extensively in this review. Another question arising is what processes could be affected by establishing a new “equilibrium” due to TWW addition? A domain of research, apart from net rates, could consider potential consequences in coupling/decoupling among nitrogen turnover processes or between nitrogen and carbon cycles, discussed below.

Moving further, both deterministic (e.g., soil moisture, pH, and redox potential) and stochastic (random) processes (dispersal limitation, ecological drift, priority effect, and microscale heterogeneity) (J. Zhou & Ning, 2017), regulating microbial assembly and function in soils (Dini‐Andreote et al., 2015; J. Wei et al., 2025), should reasonably be influenced by TWW irrigation. Distinguishing their relative contribution matters, as a potential deterministic filtering would predict TWW‐driven community shifts repeatable across studies of comparable treatment level and duration, whereas stochastic dominance would predict the site‐specific, poorly replicated outcomes that in fact characterize much of the literature synthesized in Section 4.1. This inconsistency is ambiguous evidence, however: it is equally consistent with unmeasured deterministic confounds (treatment level, duration, soil type, and plant species) as with genuine stochastic assembly, and the two cannot be disentangled without studies that hold these covariates constant while replicating across sites, a design not yet attempted in the TWW literature. Functional redundancy offers a plausible reconciliation: environmental filtering may consistently select for a function (e.g., nitrification), while which taxa are selected remains stochastically determined. This distinction has practical implications, as it determines whether findings generalize across sites and whether interventions like microbial inoculation are worth pursuing, but resolving it will require multi‐site replication under matched TWW conditions or repeated temporal sampling within a site, a design not yet applied to TWW‐irrigated systems specifically. Recently, according to a recent null‐model (iCAMP [phylogenetic‐bin‐based null model analysis for inferring community assembly mechanisms]) analysis across a Cretan watershed, the stochastic processes were found to dominate prokaryotic assembly (81%–85%) yet decouple from nitrogen‐cycling function, with a stable core community (80.6% shared taxa) persisting across 3 years despite this stochasticity (Tsiknia et al., 2026): the same reconciliation between compositional unpredictability and functional persistence proposed above, now demonstrated empirically. Consistent with the above is the example of the genetic diversity within the ammonia‐oxidizer community, which was observed to decline after long‐term (20‐year) irrigation even where function was maintained (Lopes et al., 2016).

#### 4.2.2 Water–nitrogen–carbon nexus and feedback dynamics in TWW‐irrigated soils

Typically, nitrogen and carbon cycles in soils are interlinked, driven mainly by microbial elemental stoichiometry (Spohn, 2016). The water regime regulates water availability for microbes and redox potential and acts as a carrier of various inorganic and organic compounds, regulating conditions and niches in the soil environment, which in turn can shape the soil microbiome and its activity, influencing soil biochemical processes, such as those involved in carbon and nitrogen cycles, discussed extensively in this review. From such a perspective, nitrogen–carbon–water cycles are inherently coupled in reclaimed (or even conventional) water‐receiving agroecosystems, where TWW, through water addition, irrigation frequency, wet–dry cycles, and various compounds (e.g., nitrogen, organics, and pollutant/contaminant) transfer, can act not only as the essential regulator of the nitrogen processes (demonstrated in this review study) but also as a dynamic factor of the carbon biochemistry and overall C:N stoichiometry in soil (Du et al., 2017; Yin et al., 2023; J. Zhang et al., 2021; Zhao et al., 2025), impacting further the environmental and climate performance of agroecosystem. In Figure 3, a wider conceptual framework behind the potential shift of the water–nitrogen–carbon nexus, and the feedback‐driven dynamics in agroecosystems under TWW irrigation, is described.

**FIGURE 3:**
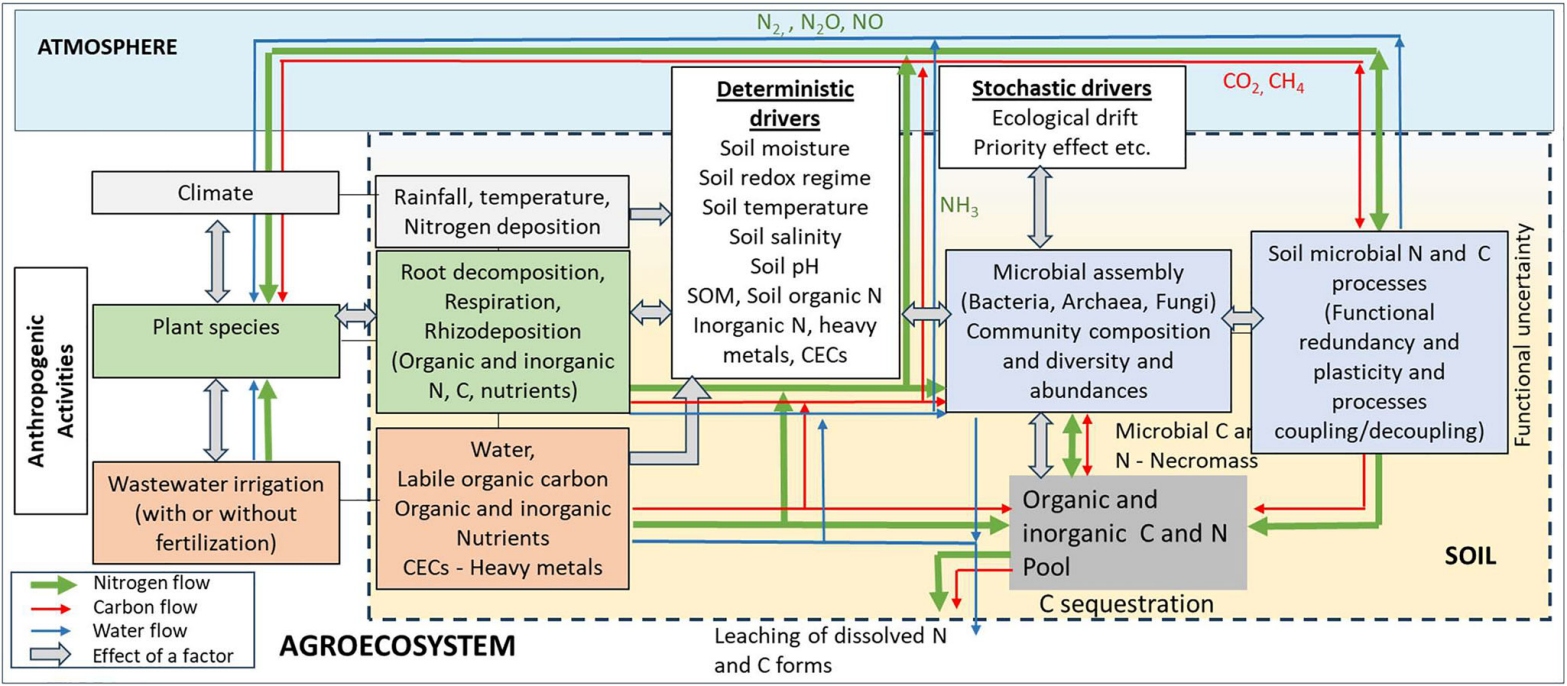
Conceptual framework illustrating the interlinkages in the water–nitrogen–carbon nexus and the feedback‐driven dynamics in an agroecosystem under treated wastewater (TWW) irrigation. According to this framework, TWW irrigation, through delivery of water, nutrients, salts, organic compounds, and contaminants of emerging concern (CECs) (dependent on treatment level in WWTPs), interacts with native soil microorganisms, soil characteristics, and plant species, regulating critical deterministic (e.g., soil moisture, redox potential, soil organic carbon, and soil pH) and stochastic drivers of the soil microbiome; This effect may influence microbial activity, plant species response, and climate feedback. In this dynamic condition, TWW, soil (with hosted microbiome), and plant species act as feedback loops controlling water, nitrogen, and carbon cycles. The figure also includes directional fluxes of aerial forms of carbon (CO_2_ and CH_4_) and nitrogen (N_2_, N_2_O, NO, and NH_3_) and leaching potential across the soil (microbiome)–plant–water–atmosphere continuum to highlight the nitrogen and carbon losses, environmental risk, and climate feedback under TWW irrigation. Colored arrows represent flows of nitrogen (green), water (blue), and carbon (red). SOM, soil organic matter.

A reasonable question could be the potential decoupling across nitrogen processes or between nitrogen and carbon processes, but so far relevant information is lacking. The literature provides examples of decoupling across various N processes (e.g., enhanced gross nitrification versus reduced gross N immobilization) due to inputs (i.e., urea) (M. Lu et al., 2021) or due to the effect of dry–wet cycles (X. Yang et al., 2024) and land use change (J. Li, Wang, et al., 2022). Potential alterations in soil microorganisms (e.g., shifts in community structure and composition) could be responsible, as discussed above. For example, loss of specific microbial taxa to specific stress was accompanied by suppressed nitrogen processes, such as potential nitrification rate and N_2_‐fixation activity (Y. Li, Ge, et al., 2021); however, decoupling between certain functions and specific taxonomic assemblages is possible under certain conditions (Q. L. Chen et al., 2020) due to the function of alternative taxa (Louca et al., 2018), also discussed above. A decoupling between carbon and nitrogen cycling has already been documented within the TWW literature reviewed here, where, in a field study, a simultaneous TWW‐driven decline in oxidizable organic carbon and DOC alongside an increase in NO_3_^−^ within the same season and soils was attributed to microbial priming of existing organic matter (Chroňáková et al., 2024).

The TWW's long‐term effects on organic carbon preservation and the underlying mechanism(s), still remain poorly examined. Due to its content, TWW can reasonably regulate organic carbon turnover processes and affect carbon stabilization mechanisms (e.g., aggregation, organo‐mineral bonding, and carbon‐necromass formation), enhancing or preventing carbon sequestration. Studies on the long‐term effects of TWW irrigation on SOC fraction partitioning (e.g., DOC, mineral‐associated organic matter [MAOM], and particulate organic matter [POM]) (Yu et al., 2022) and the mechanisms behind this partitioning may help on this task; these fractions differ in their composition and decomposition dynamics, interaction with minerals, and stability, influencing carbon sequestration potential (Lugato et al., 2021). Although no TWW study reviewed here has formally partitioned soil carbon into MAOM/POM fractions, indirect evidence of fraction‐specific responses already exists: SOM and water‐soluble DOC have been shown to respond independently to the same TWW input, with DOC alone significantly elevated while SOM remained unchanged (Adrover et al., 2012), and DOC availability has been linked to seasonal microbial enzyme activity under TWW irrigation (Elifantz et al., 2011). Finally, the potential implications of TWW irrigation on native organic matter (e.g., “priming effect”) and its role in carbon fractionation and downstream nitrogen processes remain only preliminarily explored (Chroňáková et al., 2024).

Answers to the above conceptual and knowledge gaps are of particular importance for semi‐arid and arid areas (Haddix et al., 2020), where TWW constitutes a necessity against water scarcity, and SOC preservation has become a main issue against current and emerging anthropogenic and climate stressors.

## 5 CONCLUSIONS

This review demonstrates that TWW, through delivery of a complex input in soils, including nutrients, organic carbon, and various pollutants/contaminants of concern (e.g., nitrogen and CECs), can alter soil physicochemical conditions (e.g., moisture, redox potential, pH, and salinity), reshaping soil microorganisms and their activities, and modifying critical nitrogen turnover processes (i.e., nitrogen mineralization, nitrification, and denitrification). However, no single, uniform TWW effect emerges from the studies reviewed here: the outcomes are consistently conditional on treatment level, irrigation duration, soil type, and plant species, and effects repeatedly diminish or reverse once these factors are accounted for.

Three points that deserve attention are as follows: (a) Transfer of CECs, such as antibiotics, ARGs, heavy metals, and microplastics, into soil–water–plant systems are increasingly important co‐regulator of nitrogen dynamics in TWW‐irrigated ecosystems, yet their underlying mechanisms/processes and the long‐term implications for soil functioning and health remain poorly understood; (b) it remains unknown whether soil microbial functional redundancy and plasticity persist under TWW irrigation or whether microbial communities create a new equilibrium; and (c) the complex interplay between water, nitrogen, and carbon cycles under TWW irrigation is largely unexplored. Understanding of the potential alterations in coupling across nitrogen and between nitrogen and carbon processes, SOC partitioning and stabilization, and long‐term carbon sequestration are essential to predict ecosystem response and feedback under current and emerging anthropogenic and climate stressors.

Summarizing, future work should include (a) study designs capable of disentangling TWW's co‐varying drivers rather than reporting bivariate associations; (b) long‐term field studies spanning diverse TWW qualities, soil types, and climatic conditions; (c) mechanistic, TWW‐specific investigation of CECs' effects on microorganisms involved in nitrogen cycling; and (d) predictive frameworks linking water management to coupled nitrogen and carbon outcomes.

## AUTHOR CONTRIBUTIONS

**Vasileios A. Tzanakakis**: Conceptualization; formal analysis; investigation; methodology; writing—original draft; writing—review and editing. **Georgios Giannopoulos**: Investigation; methodology; writing—review and editing. **A. G. Capodaglio**: Investigation; methodology; writing—review and editing. **A. N. Angelakis**: Investigation; methodology; writing—review and editing.

## CONFLICT OF INTEREST STATEMENT

The authors declare no conflicts of interest.
